# Supplementary material for: Predictors of exclusive breastfeeding practice among migrant and non-migrant mothers in urban China: results from a cross-sectional survey
Source: BMJ Open. 2020 Sep 6;10(9):e038268. doi: 10.1136/bmjopen-2020-038268 (PMC7476475; doi:10.1136/bmjopen-2020-038268)
Supplement: Author's manuscript [file bmjopen-2020-038268.draft_revisions.pdf]

BMJ Open

BMJ Open is committed to open peer review. As part of this commitment we make the peer review history of every article we publish publicly available.

When an article is published we post the peer reviewers' comments and the authors' responses online. We also post the versions of the paper that were used during peer review. These are the versions that the peer review comments apply to.

The versions of the paper that follow are the versions that were submitted during the peer review process. They are not the versions of record or the final published versions. They should not be cited or distributed as the published version of this manuscript.

BMJ Open is an open access journal and the full, final, typeset and author-corrected version of record of the manuscript is available on our site with no access controls, subscription charges or pay-per-view fees (http://bmjopen.bmj.com).

If you have any questions on BMJ Open's open peer review process please email info.bmjopen@bmj.com

BMJ Open

Predictors of exclusive breastfeeding practice among migrant and non-migrant mothers in China: results from a large-scale nationwide survey

Journal:	*BMJ Open*
Manuscript ID	bmjopen-2020-038268
Article Type:	Original research
Date Submitted by the Author:	06-Mar-2020

Complete List of Authors:	Li, Jia; Tsinghua University School of Social Sciences, ; China Development Research Foundation,<br>Duan, Yifan; Chinese Center for Disease Control and Prevention National Institute for Nutrition and Health<br>Bi, Ye; Chinese Center for Disease Control and Prevention National Institute for Nutrition and Health<br>Wang, Jie; Chinese Center for Disease Control and Prevention National Institute for Nutrition and Health<br>Lai, Jianqiang; Chinese Center for Disease Control and Prevention National Institute for Nutrition and Health<br>Zhao, Chen; China Development Research Foundation<br>Fang, Jin; China Development Research Foundation<br>Yang, Zhenyu; Chinese Center for Disease Control and Prevention National Institute for Nutrition and Health

Keywords:	NUTRITION & DIETETICS, PUBLIC HEALTH, EPIDEMIOLOGY

SCHOLARONE™
Manuscripts

I, the Submitting Author has the right to grant and does grant on behalf of all authors of the Work (as defined in the below author licence), an exclusive licence and/or a non-exclusive licence for contributions from authors who are: i) UK Crown employees; ii) where BMJ has agreed a CC-BY licence shall apply, and/or iii) in accordance with the terms applicable for US Federal Government officers or employees acting as part of their official duties; on a worldwide, perpetual, irrevocable, royalty-free basis to BMJ Publishing Group Ltd ("BMJ") its licensees and where the relevant Journal is co-owned by BMJ to the co-owners of the Journal, to publish the Work in this journal and any other BMJ products and to exploit all rights, as set out in our licence.

The Submitting Author accepts and understands that any supply made under these terms is made by BMJ to the Submitting Author unless you are acting as an employee on behalf of your employer or a postgraduate student of an affiliated institution which is paying any applicable article publishing charge ("APC") for Open Access articles. Where the Submitting Author wishes to make the Work available on an Open Access basis (and intends to pay the relevant APC), the terms of reuse of such Open Access shall be governed by a Creative Commons licence – details of these licences and which Creative Commons licence will apply to this Work are set out in our licence referred to above.

Other than as permitted in any relevant BMJ Author's Self Archiving Policies, I confirm this Work has not been accepted for publication elsewhere, is not being considered for publication elsewhere and does not duplicate material already published. I confirm all authors consent to publication of this Work and authorise the granting of this licence.

For peer review only

Predictors of exclusive breastfeeding practice among migrant and non-migrant mothers in China: results from a large-scale nationwide survey

Running title: Exclusive breastfeeding in urban China

Jia Li, PhD[1,2], Yifan Duan, MSc[3], Ye Bi, MSc[3], Jie Wang, PhD[3], Jianqiang Lai, PhD[3], Chen Zhao, MSocsc[2], Jin Fang, PhD[2], Zhenyu Yang, PhD[3,*]

[1] Institute of Economics, School of Social Sciences, Tsinghua University, Beijing, China

[2] China Development Research Foundation, Beijing, China

[3] National Institute for Nutrition and Health, Chinese Center for Disease Control and Prevention, Beijing, China

[*] Correspondence and reprint request to: Dr Zhenyu Yang (yangzy@ninh.chinacdc.cn), National Institute for Nutrition and Health, Chinese Center for Disease Control and Prevention, No.27 Nanwei Road, Xicheng District, Beijing, China, 100050, Phone +86-10-66237198, Fax +86-10-67791992

For peer review only

**Manuscript statistics:**	Number of tables: 3
Main text word count: 3071	Number of figures: 0
Abstract word count: 248	Number of references: 31

Disclaimers

The views expressed in the article are those of the authors and do not necessarily reflect the views of the institution and funder.

For peer review only - http://bmjopen.bmj.com/site/about/guidelines.xhtml

Funding source

The study was funded by the Bill & Melinda Gates Foundation to China Development Research Foundation (Grant Number OPP1152715).

Competing interests

None of the authors had a conflict of interest related to any part of this study or manuscript.

Authors' contributions

J.L., Y.D., Z.Y. and J.L designed the study; Y.D., Y.B., J.W. and Z.Y. supervised the field study; J.L., Y.D. and Z.Y. acquired and analyzed the data; J.L. interpreted the data and drafted the first manuscript; and Z.Y., Y.D., C.Z. and F.J. provided critical intellectual feedback to help revise the manuscript. All authors have read and approved the final manuscript.

Availability of data and materials

This dataset is available from the corresponding author on reasonable request.

Patient consent for publication

Not required

Disclosure of prior presentation of study data as an abstract or poster

None

Acknowledgements

The authors would like to thank research teams from 12 sample sites for their hard work in the data collection. The authors also want to thank all the mothers who participated in this study.

For peer review only

Abstract

Objective: To explore and compare predictors of exclusive breastfeeding (EBF) among migrant and non-migrant mothers in China.

Design: A large-scale cross-sectional study.

Setting: 12 counties/districts were covered in China.

Participants: A total number of 10408 mothers were recruited, among which a sample of 3571 mothers of 0-5 months old infants in urban China was used for analysis.

Outcome: The practice of exclusive breastfeeding was calculated based on the foods and drinks consumed in the past 24 hours as recommended by the World Health Organization.

Results: Around 30% of Chinses mothers with 0-5 months old infants practiced EBF in urban areas, with no significant difference for migrant and non-migrant mothers (*P*=0.447). Among migrant mothers, factors predictive of EBF practice included residence in big cities [AOR 1.57 (1.15 - 2.15)], premature birth [AOR 0.27 (0.09 - 0.81)], knowledge on EBF [AOR 2.01(1.52 - 2.66)], low intention of breastfeeding in the first month postpartum [AOR 0.61 (0.37 - 0.99)] and breastfeeding difficulties postpartum [AOR 1.33 (1.00 - 1.75)]. Among non-migrant mothers, in addition to similar predictors including residence in big cities [(AOR 1.46 (1.19 - 1.80)], knowledge on EBF [AOR 1.27 (1.04 - 1.55)] and low intention of breastfeeding in the first month postpartum [AOR 0.46 (0.31 - 0.69)], early initiation of breastfeeding (EIBF) [AOR 1.76 (1.35 - 2.31)] and cesarean delivery [AOR 0.74 (0.61 - 0.90)] were also factors predictive of EBF practice.

Conclusions: There was no significant difference in the prevalence of EBF between migrant and non-migrant mothers in urban China. Premature birth and breastfeeding

For peer review only - http://bmjopen.bmj.com/site/about/guidelines.xhtml

difficulties postpartum were distinctive predictors of EBF for migrants, while EIBF and cesarean delivery were unique predictors for non-migrants.

Strengths and limitations of this study

- This is the first and largest study to explore and compare breastfeeding practice and its associate factors among migrant and non-migrant mother in urban China.
- Mothers covered in this study may not be representative of mothers in urban areas since we only invited mothers who brought their infants to immunization clinics to participate, which may overestimate the prevalence of exclusive breastfeeding.
- Due to the fact that it is not a nationally representative study, the results cannot be generalized to the whole urban areas of China.

For peer review only

For peer review only - http://bmjopen.bmj.com/site/about/guidelines.xhtml

Introduction

China has witnessed rapid economic growth in the past decades. Cross-country evidence showed that breastfeeding prevalence decreased with the increase in gross domestic product per capita[1]. Similar to the global trend, the prevalence of exclusive breastfeeding (EBF) in China dropped from 27.6% in 2008 to 20.7% in 2013[2,3]. Rapid economic development is inseparable from an increasing number of domestic migrants[4]. China has witnessed an unprecedented internal migration in world history. On the one hand, better health infrastructures in cities can provide migrants with better health care services[5]. On the other hand, the household registration system and limited insurance coverage put migrants in a vulnerable situation with unfair access to urban benefits[6].

A large number of previous studies focused on immigrant mothers, who moved to another country where was different from their countries of origin[7]. Little attention has been paid to breastfeeding practice and its associated factors among migrant mothers who moved domestically. Previous literature in China disclosed that disparities in breastfeeding practice existed between urban and rural areas[3,8], employed and unemployed mothers[9], Han and other minority groups[10], high-educated and low-educated mothers[11]. Yet few studies have examined the predictors of breastfeeding practice among migrant mothers. To fill this gap of knowledge, we conducted a large-scale cross-sectional survey to examine the similarities and disparities in exclusive breastfeeding practice and its associated factors among migrant and non-migrant mothers in China.

For peer review only - http://bmjopen.bmj.com/site/about/guidelines.xhtml

Methods

Study design and participants

Data for this study was derived from a large-scale cross-sectional survey in China initiated by the China Development Research Foundation in 2017. This survey was designed by an independent team at the National Institute for Nutrition and Health of the Chinese Center for Disease Control and Prevention (NINH, China CDC). Calculation of sample size was based on the expected prevalence of EBF for 0-5 months old infants.

We adopted a multi-stage stratified cluster sampling approach when selecting the survey sample. In the first stage, all districts/counties were categorized into 4 strata, of which 12 districts/counties were selected (4 from large cities, 4 from medium and small cities, 2 from normal rural areas and 2 from poor rural areas). Selection of districts/counties considered geographic distributions, the executive capacities, and collaboration of the provincial level CDC as well as their population sizes. In the second stage, four clusters were randomly selected via probability proportional to size sampling in each selected district/county. One cluster usually had one community health center. In the last stage, mothers who brought their 0-11 months old infants to immunization clinics were randomly invited to participate in the survey until the designed sample size was met. The inclusion criteria of this study were mothers of 0-11 months old infants who signed informed consent, had no psychiatric disorders and were able to answer questions clearly. In order to decrease reporting bias of feeding practice, we only invited mothers who were the primary caregivers of their infants. In total, 10408 mothers of 0-11 months old infants were interviewed.

Patient and public involvement

For peer review only

This research was done without involvement of patients and the public.

Data collection

This survey was conducted by the NINH, China CDC in collaboration with 12 teams at the provincial level CDC from September 2017 to January 2018. Data were collected through face-to-face interviews using a structured questionnaire programmed into smartphones or tablets. The questionnaire was developed by NINH, China CDC, which consisted of eight sections including demographic characteristics, breastfeeding practice, maternal and child health, supporting environment of breastfeeding, health service, workplace, social environment and culture as well as household financial situation related factors. The study design was approved by the Medical Ethics Committee at the NINH, China CDC. We obtained written consent from all participants.

Statistical Analysis

We used the indicator recommended by the World Health Organization to assess feeding practice of 0-5 months old infants based on foods and drinks consumed in the past 24 hours. We defined infants who were fed exclusively with breastmilk in the past 24 hours as *exclusive breastfeeding*.

We first used descriptive analysis to report the selected characteristics and assess their differences between migrant and non-migrant mothers using Pearson's Chi-square test. Potential predictors were selected through reviewing relevant literature[12,13]. Chi-square tests were then used to examine the potential predictors of EBF practice for migrant and non-migrant mothers respectively.

Demographic characteristics include maternal age (<=25 years old, 26-35 years old and >=36 years old), maternal ethnicity (Han and others), maternal education

For peer review only - http://bmjopen.bmj.com/site/about/guidelines.xhtml

For peer review only

(junior high school or less, high school and college and higher education), employment type of mothers (formal and informal) and place of residence (big cities and small and medium cities). Obstetrical history consisted of parity (1 and >=2), premature birth (yes and no), low birth weight (yes and no) and mode of delivery (vaginal and cesarean). Breastfeeding-related factors included early initiation of breastfeeding (EIBF) (yes and no), knowledge on EBF (yes and no), low intention of breastfeeding within 1 month postpartum (yes and no) and breastfeeding difficulties postpartum (yes and no). Social influence denoted whether her partner, friend, mother or mother-in-law agreed that breastmilk is better than breastmilk substitutes. Health-seeking behaviors were comprised of attending antenatal visits, breastfeeding education session or mother groups.

We then performed logistic regression to identify predictors of exclusive breastfeeding practice. The selection of covariate variables was based on the p-values of Chi-square tests ($P<0.05$). Odds ratios and 95% confidence intervals were displayed. All of the data analysis was performed by STATA version 15.0 (Stata Corporation, USA). We set the level of statistical significance at $\alpha=0.05$.

For peer review only

Results

Sample description and factors associated with EBF

Considering that migrants usually concentrate in urban areas, we only focus on 3571 mothers of 0-5 months old infants who lived in big, medium and small cities. Migrant mothers were defined as those who had resided in a specific county for one month or more, and been in a place different from their registered county. We excluded 153 observations due to missing values of potential predictors, which left 3418 (>95% of the original sample) observations for statistical analysis, of which 2208 were non-migrants and 1210 were migrants. Sensitivity analysis using the full sample revealed very similar results to those shown in this study.

Table 1 displayed summary statistics and results of the bivariate analysis, reflecting significant differences in selected characteristics and associated factors of EBF practice among non-migrant and migrant mothers. The prevalence of EBF was 30.3% among non-migrant mothers, which was not statistically different from 29.0% of migrant mothers (P=0.447).

More than 60% of mothers were between 26-35 years old in both groups, while the proportion of mothers above 36 years old was significantly higher in non-migrant mothers. The likelihood of being Han ethnicity and being formally employed was statistically higher in non-migrant mothers than migrant mothers as well (Table 1). A statistically higher rate of migrant mothers lived in big cities than non-migrant mothers (P<0.001). For non-migrant mothers, demographic characteristics like maternal education, employment status of mothers and place of residence were significantly associated with EBF (P<0.001). For migrant mothers, in addition to maternal education, employment status of mothers and place of residence, the ethnicity of mothers also significantly affected the prevalence of EBF. Mothers with a college or higher

For peer review only - http://bmjopen.bmj.com/site/about/guidelines.xhtml

education, who were formally employed and lived in big cities were more likely to practice EBF in both groups. Mothers from the Han ethnic group were more likely to practice EBF than mothers from minority groups among the migrant group (Table 1).

Among factors about obstetrical history, the percentage of having only one child among migrant mothers was statistically higher than non-migrant mothers (P<0.001). The proportions of premature birth and low birth weight were quite similar between migrant and non-migrant mothers, while the rate of cesarean section was statistically higher in non-migrant mothers than migrant mothers. Cesarean section significantly decreased the likelihood of EBF for both migrant and non-migrant mothers. However, for migrant women, premature birth and low birth weight of infants also negatively associated with EBF.

EIBF, having a low intention of breastfeeding within 1 month postpartum and breastfeeding difficulties postpartum occurred more frequently among migrant mothers than non-migrant mothers, while only the prevalence of having breastfeeding difficulties postpartum was statistically different between the two groups. Unlike the non-migrant group where EIBF associated with EBF significantly (P<0.001), migrant mothers were more likely to practice EBF if they had breastfeeding difficulties postpartum. More than half of the mothers knew the definition of EBF, and around 10 percent of them had a low intention of breastfeeding within 1 month postpartum in both groups. Mothers in both groups were more likely to practice EBF if they know the definition of it and were less likely to do it if their intentions of breastfeeding were low within the 1 month postpartum.

Even though more than 80% of mothers received positive influence from their partners, friends, mothers or mothers-in-law on the fact that breastmilk is better than breastmilk substitutes, these influences differed among migrant and non-migrant

For peer review only - http://bmjopen.bmj.com/site/about/guidelines.xhtml

mothers. The influence of partner and friend was significantly associated with EBF for non-migrant mothers, while the influence of partner and their own mothers were significantly associated with EBF for migrant mothers.

Non-migrant mothers were more likely to adopt health-seeking behaviors like attending antenatal visits, breastfeeding education sessions and mother groups than migrant mothers. However, only the proportion of mothers who attended antenatal visits was statistically different between these two groups. Attending breastfeeding education sessions only significantly affected the EBF practice of non-migrant mothers.

Predictors of breastfeeding practice among migrant and non-migrant mothers

We then performed logistic regression to determine the predictors of exclusive breastfeeding respectively. We only kept covariates with *p*-values smaller than 0.05 in Table 1.

Among demographic factors, place of residence was significantly associated with EBF. Migrants living in big cities were more likely to practice EBF (adjusted OR(AOR) =1.57, 95% CI 1.15 - 2.15) after adjusting for other potential confounders. The odds of EBF were lower among migrant mothers with preterm infants (AOR= 0.27, 95% CI 0.09 - 0.81) and having a low intention of breastfeeding in the first month postpartum (AOR= 0.61, 95% CI 0.37 - 0.99). Migrant mothers who knew EBF (AOR= 2.01, 95% CI 1.52 - 2.66) and who encountered breastfeeding difficulties postpartum (AOR= 1.33, 95% CI 1.00 - 1.75) were more likely to practice EBF (Table 2).

Among non-migrant mothers, we found that residence in big cities (AOR=1.46, 95% CI 1.19 - 1.80), practicing EIBF (AOR=1.76, 95% CI 1.35 - 2.31) and knowledge on EBF (AOR=1.27, 95% CI 1.04 - 1.55) were significantly associated with optimal breastfeeding practice. However, cesarean section (AOR=0.74, 95% CI 0.61 - 0.90)

For peer review only - http://bmjopen.bmj.com/site/about/guidelines.xhtml

and low intention of breastfeeding (AOR=0.46, 95% CI 0.31 - 0.69) significantly decreased the likelihood to practice EBF (Table 3).

For peer review only

For peer review only - http://bmjopen.bmj.com/site/about/guidelines.xhtml

Discussion

In this study, we found that breastfeeding practice was suboptimal in both migrant and non-migrant mothers. The prevalence of EBF among non-migrant women was not significantly different from that of migrant mothers. This is the first and largest study to explore and compare breastfeeding practice and their potential predictors among migrant and non-migrant mothers in China. Multivariate regression results suggested that similar predictors of EBF for mothers in both groups included residence in big cities, knowledge on EBF and low intention of breastfeeding in the first month postpartum. Mothers in big cities were approximately 1.5 times more likely to practice EBF. Among migrant mothers, having premature infants and breastfeeding difficulties postpartum were associated with EBF practice. However, for non-migrant mothers, EIBF and cesarean delivery were unique predictors of EBF practice.

Living in big cities was the only common variable in demographic characteristics that were significantly associated with EBF practice in both groups. Previous literature also suggested a higher rate of EBF in big cities than in middle and small cities in China using nationally representative data derived from the Chinese National Nutrition and Health Survey conducted in 2013[3]. This is possibly due to better knowledge and supporting environment for mothers in big cities than those living in small and medium cities regardless of their migration status. For example, the proportion of mothers who received information on encouraging breastfeeding was higher in big cities of 86.7% than that in small and medium cities of 76.1%, and more than 90% of them received encouragement from the delivery hospital in big cities. This revealed that both migrant and non-migrant mothers may benefit from better breastfeeding-related education and support in big cities in China.

Another common predictor of EBF practice for both migrant and non-migrant mothers was knowledge of EBF. Some of the previous studies used knowledge scores on breastfeeding benefits as a predictor of breastfeeding practice, but they failed to provide consistent evidence

For peer review only - http://bmjopen.bmj.com/site/about/guidelines.xhtml

on its association with EBF[8,14]. However, we only focused on the key message that infants should be exclusively breastfed within 6 months. This implies that future education intervention projects should pay more attention to disseminating core messages in promoting optimal breastfeeding practice.

Additionally, we also found that the low intention of breastfeeding in the first month postpartum significantly decreased the odds of practicing EBF for both groups. In alignment with previous studies, nipple pain or fissure within the first month postpartum was the commonest reason for the low intention of breastfeeding among mothers regardless of their migration status[15,16]. Given that nipple pain was often attributed to incorrect positioning and attachment[16], health care providers need to find effective measures to help to establish proper positioning of mothers, good attachment of the babies to the breast as well as effective sucking.

In addition to common predictors of EBF practice explained above, there were unique risk factors for migrant and non-migrant mothers respectively.

Among migrant mothers, having premature infants significantly decreased the prevalence of EBF. The EBF rate for migrant mothers with preterm infants was 9.8%, which was largely lower than non-migrant mothers of 31.0%. The low EBF rate can be attributable to mother-infant separation in neonatal intensive care units (NICUs) in China[17], which resulted in many difficulties and challenges for mothers with premature infants. The disparity in the EBF rate of premature infants may come from different choices of hospitalization services between migrant and mon-migrant mothers. A recent study in China found that migrants especially those from rural areas were less likely to use tertiary hospitals, where well-trained health workers and good service were concentrated[18].

Different from previous studies, we failed to find that breastfeeding difficulties were barriers to optimal breastfeeding practice[19]. In contrast, migrant mothers who encountered

For peer review only - http://bmjopen.bmj.com/site/about/guidelines.xhtml

breastfeeding difficulties were more likely to breastfeed their infants exclusively in our sample. This is possibly because around 95% of the breastfeeding difficulties encountered by migrant mothers were solved successfully, and over 80% of them felt that these difficulties didn't impede future breastfeeding. A systematic review of previous literature suggested that the combination of peer support and professional support can effectively ensure the continuation of breastfeeding[20]. Experience in developed countries suggested that health professionals such as midwives, health visitors as well as lactation consultants had started to get involved in peer support programs collaborating with voluntary breastfeeding organizations[21]. The government may take the initiative to fund breastfeeding support projects and provide incentives to health professionals to actively provide support to lactating mothers.

Among non-migrant mothers, EIBF and cesarean section were distinctive predictors of EBF. The rate of EIBF was 12.8% in non-migrant mothers, which was a little bit higher than a study in Sichuan province of 9%[22], but much lower than the national prevalence in 2013 of 28.7%[23] and a study in central and western China of 59.4%[24]. A large regional disparity of EIBF in China may be due to the delayed process of implementing Early Essential Newborn Care (EENC) in China. Even in places where EENC had been implemented, skin-to-skin contact was often interrupted due to inadequate facility resources and early initiation of breastfeeding was difficult to be ensured[25].

For obstetrical history-related factors, we also found that the prevalence of cesarean delivery was high and negatively associated with EBF among non-migrant mothers only. The prevalence of cesarean delivery was 39.7% among non-migrant mothers, which was higher than the national average in 2018 of 36.7%[26]. Even though China had made efforts in decreasing cesarean delivery in the past decade[27], the national prevalence is still far higher than the suggested prevalence of 10-15% by the World Health Organization[28]. The government and health facilities need to make effective policies and measures in addressing

For peer review only - http://bmjopen.bmj.com/site/about/guidelines.xhtml

non-clinical reasons for preferring cesarean delivery such as maternal request and perceived convenience[29].

This study derived data from a large-scale cross-sectional survey covering 8 sample sites in urban areas, which is the first and largest study to explore and compare breastfeeding practice and their potential predictors among migrant and non-migrant mothers in China. Findings in this study filled the knowledge gap in the similarities and differences in the predictors of EBF prevalence among migrant and non-migrant mothers, which had important implications for future studies as well as health interventions to promote optimal breastfeeding practice in China.

However, our study still faced the following limitations. First, differences in predictors of EBF among migrant and non-migrant mothers revealed in this study may come from different choices of hospitalization services. Migrant mothers may have restricted access to better health services due to social exclusions and lower social-economic status. Future studies need to have a closer look at the differences in health facility choice among migrant and non-migrant mothers and identify potential obstacles faced by migrants. Second, mothers covered in this study may not be representative of the mothers in general. That is because we only interviewed mothers who brought their infants into immunization clinics at the time of the interview, which naturally excluded children who were left behind in their early childhood and may overestimate the prevalence of exclusive breastfeeding for both groups [30,31]. Third, when selecting sample sites in the first stage, we considered the executive capacities, and collaboration of the provincial level CDC. Thus, sample sites selected in this study to some extent reflected a higher efficiency of health systems at the country/district level. Caution is needed when interpreting these results and results disclosed in this study cannot be generalized to the whole urban areas of China.

For peer review only - http://bmjopen.bmj.com/site/about/guidelines.xhtml

To conclude, this study found that there was no significant difference in the prevalence of EBF between migrant and non-migrant mothers in our sampled urban areas of China. Even though the predictors of EBF shared some similarities between migrant and non-migrant mothers, future interventions may still need to adopt different strategies in promoting optimal breastfeeding practice in different groups.

For peer review only

For peer review only - http://bmjopen.bmj.com/site/about/guidelines.xhtml

Reference

1 Victora CG, Bahl R, Barros AJD, *et al.* Breastfeeding in the 21st century: Epidemiology, mechanisms, and lifelong effect. Lancet. 2016. doi:10.1016/S0140-6736(15)01024-7

2 Center for Health Statistics and Information at Ministry of Health of People's Republic of China. *An Analysis Report of National Health Services Survey in China,2008*. Beijing: 2009.

3 Duan Y, Yang Z, Lai J, *et al.* Exclusive breastfeeding rate and complementary feeding indicators in China: A national representative survey in 2013. *Nutrients* Published Online First: 2018. doi:10.3390/nu10020249

4 Wang XR, Hui ECM, Sun JX. Population migration, urbanization and housing prices: Evidence from the cities in China. *Habitat Int* Published Online First: 2017. doi:10.1016/j.habitatint.2017.05.010

5 Song Y, Sun W. Health Consequences of Rural-to-Urban Migration: Evidence from Panel Data in China. *Health Econ* Published Online First: 2016. doi:10.1002/hec.3212

6 Peng Y, Chang W, Zhou H, *et al.* Factors associated with health-seeking behavior among migrant workers in Beijing, China. *BMC Health Serv Res* Published Online First: 2010. doi:10.1186/1472-6963-10-69

7 Dennis CL, Shiri R, Brown HK, *et al.* Breastfeeding rates in immigrant and non-immigrant women: A systematic review and meta-analysis. Matern. Child Nutr. 2019. doi:10.1111/mcn.12809

8 Gao H, Wang Q, Hormann E, *et al.* Breastfeeding practices on postnatal wards in urban and rural areas of the Deyang region, Sichuan province of China. *Int Breastfeed J* Published Online First: 2016. doi:10.1186/s13006-016-0070-0

For peer review only

For peer review only - http://bmjopen.bmj.com/site/about/guidelines.xhtml

9 Li L, Li S, Ali M, *et al.* Feeding practice of infants and their correlates in urban areas of Beijing, China. *Pediatr Int* Published Online First: 2003. doi:10.1046/j.1442-200X.2003.01754.x

10 Xu F, Binns C, Nazi G, *et al.* A comparison of breastfeeding among Han, Uygur and other ethnic groups in Xinjiang, PR China. *BMC Public Health* Published Online First: 2006. doi:10.1186/1471-2458-6-196

11 Zhao J, Zhao Y, Du M, *et al.* Maternal education and breastfeeding practices in China: A systematic review and meta-analysis. Midwifery. 2017. doi:10.1016/j.midw.2017.03.011

12 Rollins NC, Bhandari N, Hajeebhoy N, *et al.* Why invest, and what it will take to improve breastfeeding practices? Lancet. 2016. doi:10.1016/S0140-6736(15)01044-2

13 Dennis CL, Gagnon A, Van Hulst A, *et al.* Prediction of duration of breastfeeding among migrant and canadian-born women: Results from a multi-center study. *J Pediatr* Published Online First: 2013. doi:10.1016/j.jpeds.2012.06.041

14 Shi L, Zhang J, Wang Y, *et al.* Breastfeeding in rural China: Association between knowledge, attitudes, and practices. *J Hum Lact* Published Online First: 2008. doi:10.1177/0890334408319157

15 McCann MF, Baydar N, Williams RL. Breastfeeding attitudes and reported problems in a national sample of WIC participants. *J Hum Lact* Published Online First: 2007. doi:10.1177/0890334407307882

16 Kent JC, Ashton E, Hardwick CM, *et al.* Nipple pain in breastfeeding mothers: Incidence, causes and treatments. *Int J Environ Res Public Health* Published Online First: 2015. doi:10.3390/ijerph121012247

For peer review only

17 Wang Y, Briere CE, Xu W, *et al.* Factors Affecting Breastfeeding Outcomes at Six Months in Preterm Infants. *J Hum Lact* Published Online First: 2019. doi:10.1177/0890334418771307

18 Wang H, Zhang D, Hau Z, *et al.* Association between social health insurance and choice of hospitals among internal migrants in China: A national cross-sectional study. *BMJ Open* Published Online First: 2018. doi:10.1136/bmjopen-2017-018440

19 Odom EC, Li R, Scanlon KS, *et al.* Reasons for earlier than desired cessation of breastfeeding. *Pediatrics* Published Online First: 2013. doi:10.1542/peds.2012-1295

20 Kaunonen M, Hannula L, Tarkka MT. A systematic review of peer support interventions for breastfeeding. J. Clin. Nurs. 2012. doi:10.1111/j.1365-2702.2012.04071.x

21 Dykes F. Government funded breastfeeding peer support projects: Implications for practice. *Matern Child Nutr* Published Online First: 2005. doi:10.1111/j.1740-8709.2004.00006.x

22 Tang L, Binns CW, Lee AH, *et al.* Low Prevalence of Breastfeeding Initiation within the First Hour of Life in a Rural Area of Sichuan Province, China. *Birth* Published Online First: 2013. doi:10.1111/birt.12038

23 UNICEF. Children in China: An Atlas of Social Indicators of Children in China 2018. Beijing: 2018.

24 Guo S, Fu X, Scherpbier RW, *et al.* Breastfeeding rates in central and western China in 2010: implications for child and population health. *Bull World Health Organ* Published Online First: 2013. doi:10.2471/BLT.12.111310

25 Qu W, Yue Q, Wang Y, *et al.* Implementation of the Early Essential Newborn

For peer review only - http://bmjopen.bmj.com/site/about/guidelines.xhtml

Care (EENC) on neonatal outcomes in west China: an observational study. *Lancet* Published Online First: 2018. doi:10.1016/s0140-6736(18)32683-7

26 Li H, Hellerstein S, Zhou Y, *et al.* Trends in Cesarean Delivery Rates in China, 2008-2018. *JAMA* 2020;**323**:89–91. doi:10.1001/jama.2019.17595

27 Liang J, Mu Y, Li X, *et al.* Relaxation of the one child policy and trends in caesarean section rates and birth outcomes in China between 2012 and 2016: Observational study of nearly seven million health facility births. *BMJ* Published Online First: 2018. doi:10.1136/bmj.k817

28 Betran AP, Torloni MR, Zhang JJ, *et al.* WHO statement on caesarean section rates. BJOG An Int. J. Obstet. Gynaecol. 2016. doi:10.1111/1471-0528.13526

29 Long Q, Kingdon C, Yang F, *et al.* Prevalence of and reasons for women's, family members', and health professionals' preferences for cesarean section in China: A mixed-methods systematic review. *PLoS Med* Published Online First: 2018. doi:10.1371/journal.pmed.1002672

30 Tian X, Ding C, Shen C, *et al.* Does parental migration have negative impact on the growth of left-behind children?—new evidence from longitudinal data in rural China. *Int J Environ Res Public Health* Published Online First: 2017. doi:10.3390/ijerph14111308

31 Zhang N, Bécares L, Chandola T. Does the timing of parental migration matter for child growth? A life course study on left-behind children in rural China. *BMC Public Health* 2015;**15**:966. doi:10.1186/s12889-015-2296-y

For peer review only - http://bmjopen.bmj.com/site/about/guidelines.xhtml

Table 1 Factors associated with EBF practice among mothers of 0-5 months old infants by migration status

	Non-migrant women(n=2208)					Migrant women (n=1210)					
	Total		EBF		*P*-value[b]	Total		EBF		*P*-value[b]	*P*-value[a]
	N	%	n	%		N	%	n	%		
Demographic characteristics											
Maternal age											
<=25 years old	451	20.4	129	28.6	0.691	287	23.7	84	29.3	0.534	<0.001
26-35 years old	1417	64.2	434	30.6		805	66.5	238	29.6		
36+ years old	340	15.4	105	30.9		118	9.8	29	24.6		
Maternal ethnicity											
Others	360	16.3	123	34.2	0.077	251	20.7	57	22.7	0.014	0.001
Han	1848	83.7	545	29.5		959	79.3	294	30.7		
Maternal education											
Junior high school or less	666	30.2	167	25.1	<0.001	364	30.1	80	22.0	<0.001	0.471
High school	424	19.2	106	25.0		213	17.6	52	24.4		
College or higher education	1118	50.6	395	35.3		633	52.3	219	34.6		
Mother was formally employed											
No	1359	61.5	366	26.9	<0.001	794	65.6	199	25.1	<0.001	0.018
Yes	849	38.5	302	35.6		416	34.4	152	36.5		
Place of residence											
Small and medium sized cities	1225	55.5	299	24.4	<0.001	413	34.1	83	20.1	<0.001	<0.001
Big cities	983	44.5	369	37.5		797	65.9	268	33.6		
Obstetrical history											
Parity											
>=2	1184	53.6	357	30.2	0.911	512	42.3	135	26.4	0.083	<0.001
1	1024	46.4	311	30.4		698	57.7	216	30.9		
Premature birth											
No	2121	96.1	641	30.2	0.871	1169	96.6	347	29.7	0.006	0.416
Yes	87	3.9	27	31.0		41	3.4	4	9.8		
Low birthweight											
No	2113	95.7	643	30.4	0.393	1155	95.5	343	29.7	0.016	0.740
Yes	95	4.3	25	26.3		55	4.5	8	14.5		
Mode of delivery											
Vaginal	1332	60.3	439	33.0	0.001	784	64.8	246	31.4	0.014	0.010
Cesarean	876	39.7	229	26.1		426	35.2	105	24.6		
Breastfeeding-related factors											
EIBF											
No	1925	87.2	540	28.1	<0.001	1037	85.7	291	28.1	0.076	0.223
Yes	283	12.8	128	45.2		173	14.3	60	34.7		

For peer review only - http://bmjopen.bmj.com/site/about/guidelines.xhtml

Know EBF											
No	945	42.8	238	25.2	<0.001	510	42.1	99	19.4	<0.001	0.713
Yes	1263	57.2	430	34.0		700	57.9	252	36.0		
Low intention of breastfeeding within 1 month postpartum											
No	2027	91.8	637	31.4	<0.001	1091	90.2	326	29.9	0.043	0.106
Yes	181	8.2	31	17.1		119	9.8	25	21.0		
Breastfeeding difficulties postpartum											
No	1573	71.2	468	29.8	0.419	815	67.4	211	25.9	0.001	0.018
Yes	635	28.8	200	31.5		395	32.6	140	35.4		
Social influence											
Partner supports breastfeeding											
No	270	12.2	64	23.7	0.012	145	12.0	31	21.4	0.031	0.834
Yes	1938	87.8	604	31.2		1065	88.0	320	30.0		
Friend supports breastfeeding											
No	318	14.4	80	25.2	0.032	182	15.0	46	25.3	0.229	0.613
Yes	1890	85.6	588	31.1		1028	85.0	305	29.7		
Grandmother supports breastfeeding											
No	256	11.6	71	27.7	0.351	155	12.8	32	20.6	0.014	0.296
Yes	1952	88.4	597	30.6		1055	87.2	319	30.2		
Mother in law supports breastfeeding											
No	313	14.2	93	29.7	0.822	148	12.2	36	24.3	0.180	0.112
Yes	1895	85.8	575	30.3		1062	87.8	315	29.7		
Health-seeking behaviors											
Attend antenatal visits											
No	89	4.0	27	30.3	0.986	65	5.4	23	35.4	0.244	0.071
Yes	2119	96.0	641	30.3		1145	94.6	328	28.6		
Attend breastfeeding education sessions											
No	1103	50.0	312	28.3	0.044	619	51.2	171	27.6	0.278	0.501
Yes	1105	50.0	356	32.2		591	48.8	180	30.5		
Attend mother groups											
No	1377	62.4	436	31.7	0.063	777	64.2	225	29.0	0.958	0.284
Yes	831	37.6	232	27.9		433	35.8	126	29.1		

[a] *P*-value of chi-square test to compare distribution of covariate variables between migrant and non-migrant mothers.

[b] *P*-value of chi-square test to identify risk factors of exclusive breastfeeding practice among migrant and non-migrant mothers respectively.

For peer review only - http://bmjopen.bmj.com/site/about/guidelines.xhtml

Table 2 Predictors of EBF practice among migrant mothers of 0-5 months old infants (n=1210)

Risk factors	UOR[a]	95% CI	AOR[b]	95% CI
Maternal ethnicity				
Others	1.0		1.0	
Han	1.02	(0.84 - 1.24)	1.14	(0.80 - 1.63)
Maternal education				
Junior high school or less	1.0		1.0	
High school	1.05	(0.83 - 1.32)	0.97	(0.64 - 1.48)
College or higher education	1.71***	(1.44 - 2.04)	1.26	(0.86 - 1.83)
Mather was formally employed				
No	1.0		1.0	
Yes	1.57***	(1.35 - 1.83)	1.28	(0.93 - 1.76)
Place of residence				
Small and medium cities	1.0		1.0	
Big cities	1.83***	(1.58 - 2.13)	1.57**	(1.15 - 2.15)
Premature birth				
No	1.0		1.0	
Yes	0.74	(0.49 - 1.12)	0.27*	(0.09 - 0.81)
Low birthweight				
No	1.0		1.0	
Yes	0.65*	(0.44 - 0.97)	0.65	(0.29 - 1.49)
Mode of delivery				
Vaginal	1.0		1.0	
Cesarean	0.72***	(0.62 - 0.84)	0.81	(0.61 - 1.08)
Knowledge on EBF				
No	1.0		1.0	
Yes	1.77***	(1.52 - 2.06)	2.01***	(1.52 - 2.66)
Low intention of breastfeeding within 1 month postpartum				
No	1.0		1.0	
Yes	0.51***	(0.38 - 0.69)	0.61*	(0.37 - 0.99)
Breastfeeding difficulties postpartum				

For peer review only

No	1.0		1.0	
Yes	1.24**	(1.06 - 1.45)	1.33*	(1.00 - 1.75)
Father supports breastfeeding				
No	1.0		1.0	
Yes	1.50**	(1.18 - 1.91)	1.34	(0.77 - 2.31)
Grandmother supports breastfeeding				
No	1.0		1.0	
Yes	1.31*	(1.03 - 1.66)	1.51	(0.89 - 2.58)

[a] Unadjusted odds ratios. [b] Odds ratios were adjusted for all the covariates in the model. * $P < 0.05$, ** $P < 0.01$, *** $P < 0.001$.

For peer review only - http://bmjopen.bmj.com/site/about/guidelines.xhtml

Table 3 Predictors of EBF practice among non-migrant mothers of 0-5 months old infants (n=2208)

Risk factors	UOR[a]	95% CI	AOR[b]	95% CI
Maternal education				
Junior high school or less	1.0		1.0	
High school	1.05	(0.83 - 1.32)	0.93	(0.70 - 1.25)
College or higher education	1.71***	(1.44 - 2.04)	1.23	(0.92 - 1.64)
Mother was formally employed				
No	1.0		1.0	
Yes	1.57***	(1.35 - 1.83)	1.11	(0.86 - 1.43)
Place of residence				
Small and medium cities	1.0		1.0	
Big cities	1.83***	(1.58 - 2.13)	1.46***	(1.19 - 1.80)
Mode of delivery				
Vaginal	1.0		1.0	
Cesarean	0.72***	(0.62 - 0.84)	0.74**	(0.61 - 0.90)
EIBF				
No	1.0		1.0	
Yes	1.80***	(1.47 - 2.20)	1.76***	(1.35 - 2.31)
Knowledge on EBF				
No	1.0		1.0	
Yes	1.77***	(1.52 - 2.06)	1.27*	(1.04 - 1.55)
Low intention of breastfeeding within 1 month postpartum				
No	1.0		1.0	
Yes	0.51***	(0.38 - 0.69)	0.46***	(0.31 - 0.69)
Father supports breastfeeding				
No	1.0		1.0	
Yes	1.50**	(1.18 - 1.91)	1.21	(0.84 - 1.77)
Friend supports breastfeeding				
No	1.0		1.0	
Yes	1.31*	(1.05 - 1.63)	1.13	(0.80 - 1.59)
Attend breastfeeding education session				
No	1.0		1.0	
Yes	1.19*	(1.02 - 1.37)	1.12	(0.92 - 1.36)

[a] Unadjusted odds ratios. [b] Odds ratios were adjusted for all the covariates in the model. * $P < 0.05$, ** $P < 0.01$, *** $P < 0.001$.

For peer review only - http://bmjopen.bmj.com/site/about/guidelines.xhtml
For peer review only

STROBE Statement—Checklist of items that should be included in reports of ***cross-sectional studies***

	Item No	Page number in manuscript	Recommendation
**Title and abstract**	1	Page 3	(*a*) Indicate the study's design with a commonly used term in the title or the abstract
		Page 3	(*b*) Provide in the abstract an informative and balanced summary of what was done and what was found
**Introduction**			
Background/rationale	2	Page 5	Explain the scientific background and rationale for the investigation being reported
Objectives	3	Page 5	State specific objectives, including any prespecified hypotheses
**Methods**			
Study design	4	Page 6	Present key elements of study design early in the paper
Setting	5	Page 6-7	Describe the setting, locations, and relevant dates, including periods of recruitment, exposure, follow-up, and data collection
Participants	6	Page 6	(*a*) Give the eligibility criteria, and the sources and methods of selection of participants
Variables	7	Page 7	Clearly define all outcomes, exposures, predictors, potential confounders, and effect modifiers. Give diagnostic criteria, if applicable
Data sources/ measurement	8*	Page 7	For each variable of interest, give sources of data and details of methods of assessment (measurement). Describe comparability of assessment methods if there is more than one group
Bias	9	Page 6	Describe any efforts to address potential sources of bias
Study size	10	Page 6	Explain how the study size was arrived at
Quantitative variables	11	Page 7-8	Explain how quantitative variables were handled in the analyses. If applicable, describe which groupings were chosen and why
Statistical methods	12	Page 7-8	(*a*) Describe all statistical methods, including those used to control for confounding
		Page 7-8	(*b*) Describe any methods used to examine subgroups and interactions
		Page 9	(*c*) Explain how missing data were addressed
		Not applicable	(*d*) If applicable, describe analytical methods taking account of sampling strategy
		Page 9	(*e*) Describe any sensitivity analyses
**Results**			
Participants	13*	Page 9	(a) Report numbers of individuals at each stage of study—eg numbers potentially eligible, examined for eligibility, confirmed eligible, included in the study, completing follow-up, and analysed
		Not applicable	(b) Give reasons for non-participation at each stage
		Not applicable	(c) Consider use of a flow diagram
Descriptive data	14*	Page 9	(a) Give characteristics of study participants (eg demographic, clinical, social) and information on exposures and potential confounders
		Not applicable	(b) Indicate number of participants with missing data for each variable of interest

For peer review only - http://bmjopen.bmj.com/site/about/guidelines.xhtml

Outcome data	15*	Page 9	Report numbers of outcome events or summary measures
Main results	16	Page 23-25	(*a*) Give unadjusted estimates and, if applicable, confounder-adjusted estimates and their precision (eg, 95% confidence interval). Make clear which confounders were adjusted for and why they were included
		Not applicable	(*b*) Report category boundaries when continuous variables were categorized
		Not applicable	(*c*) If relevant, consider translating estimates of relative risk into absolute risk for a meaningful time period
Other analyses	17	Page 21	Report other analyses done—eg analyses of subgroups and interactions, and sensitivity analyses
**Discussion**			
Key results	18	Page 13	Summarise key results with reference to study objectives
Limitations	19	Page 16	Discuss limitations of the study, taking into account sources of potential bias or imprecision. Discuss both direction and magnitude of any potential bias
Interpretation	20	Page 16	Give a cautious overall interpretation of results considering objectives, limitations, multiplicity of analyses, results from similar studies, and other relevant evidence
Generalisability	21	Page 16	Discuss the generalisability (external validity) of the study results
**Other information**			
Funding	22	Title page	Give the source of funding and the role of the funders for the present study and, if applicable, for the original study on which the present article is based

*Give information separately for exposed and unexposed groups.

Note: An Explanation and Elaboration article discusses each checklist item and gives methodological background and published examples of transparent reporting. The STROBE checklist is best used in conjunction with this article (freely available on the Web sites of PLoS Medicine at http://www.plosmedicine.org/, Annals of Internal Medicine at http://www.annals.org/, and Epidemiology at http://www.epidem.com/). Information on the STROBE Initiative is available at www.strobe-statement.org.

For peer review only

For peer review only - http://bmjopen.bmj.com/site/about/guidelines.xhtml

BMJ Open

Predictors of exclusive breastfeeding practice among migrant and non-migrant mothers in urban China: results from a cross-sectional survey

Journal:	*BMJ Open*
Manuscript ID	bmjopen-2020-038268.R1
Article Type:	Original research
Date Submitted by the Author:	15-May-2020
Complete List of Authors:	Li, Jia; Tsinghua University School of Social Sciences, ; China Development Research Foundation,<br>Duan, Yifan; Chinese Center for Disease Control and Prevention National Institute for Nutrition and Health<br>Bi, Ye; Chinese Center for Disease Control and Prevention National Institute for Nutrition and Health<br>Wang, Jie; Chinese Center for Disease Control and Prevention National Institute for Nutrition and Health<br>Lai, Jianqiang; Chinese Center for Disease Control and Prevention National Institute for Nutrition and Health<br>Zhao, Chen; China Development Research Foundation<br>Fang, Jin; China Development Research Foundation<br>Yang, Zhenyu; Chinese Center for Disease Control and Prevention National Institute for Nutrition and Health
**Primary Subject Heading**:	Public health
Secondary Subject Heading:	Epidemiology
Keywords:	NUTRITION & DIETETICS, PUBLIC HEALTH, EPIDEMIOLOGY

SCHOLARONE™
Manuscripts

I, the Submitting Author has the right to grant and does grant on behalf of all authors of the Work (as defined in the below author licence), an exclusive licence and/or a non-exclusive licence for contributions from authors who are: i) UK Crown employees; ii) where BMJ has agreed a CC-BY licence shall apply, and/or iii) in accordance with the terms applicable for US Federal Government officers or employees acting as part of their official duties; on a worldwide, perpetual, irrevocable, royalty-free basis to BMJ Publishing Group Ltd ("BMJ") its licensees and where the relevant Journal is co-owned by BMJ to the co-owners of the Journal, to publish the Work in this journal and any other BMJ products and to exploit all rights, as set out in our licence.

The Submitting Author accepts and understands that any supply made under these terms is made by BMJ to the Submitting Author unless you are acting as an employee on behalf of your employer or a postgraduate student of an affiliated institution which is paying any applicable article publishing charge ("APC") for Open Access articles. Where the Submitting Author wishes to make the Work available on an Open Access basis (and intends to pay the relevant APC), the terms of reuse of such Open Access shall be governed by a Creative Commons licence – details of these licences and which Creative Commons licence will apply to this Work are set out in our licence referred to above.

Other than as permitted in any relevant BMJ Author's Self Archiving Policies, I confirm this Work has not been accepted for publication elsewhere, is not being considered for publication elsewhere and does not duplicate material already published. I confirm all authors consent to publication of this Work and authorise the granting of this licence.

For peer review only

Predictors of exclusive breastfeeding practice among migrant and non-migrant mothers in urban China: results from a cross-sectional survey

Running title: Exclusive breastfeeding in urban China

Jia Li, PhD[1,2], Yifan Duan, MSc[3], Ye Bi, MSc[3], Jie Wang, PhD[3], Jianqiang Lai, PhD[3], Chen Zhao, MSocsc[2], Jin Fang, PhD[2], Zhenyu Yang, PhD[3,*]

[1] Institute of Economics, School of Social Sciences, Tsinghua University, Beijing, China

[2] China Development Research Foundation, Beijing, China

[3] National Institute for Nutrition and Health, Chinese Center for Disease Control and Prevention, Beijing, China

[*] Correspondence and reprint request to: Dr Zhenyu Yang (yangzy@ninh.chinacdc.cn), National Institute for Nutrition and Health, Chinese Center for Disease Control and Prevention, No.27 Nanwei Road, Xicheng District, Beijing, China, 100050, Phone +86-10-66237198, Fax +86-10-67791992

For peer review only

**Manuscript statistics:**	Number of tables: 3
Main text word count: 3609	Number of figures: 0
Abstract word count: 277	Number of references: 38

Disclaimers

The views expressed in the article are those of the authors and do not necessarily reflect the views of the institution and funder.

For peer review only - http://bmjopen.bmj.com/site/about/guidelines.xhtml

Funding source

The study was funded by the Bill & Melinda Gates Foundation to China Development Research Foundation (Grant Number OPP1152715).

Competing interests

None of the authors had a conflict of interest related to any part of this study or manuscript.

Authors' contributions

J.L., Y.D., Z.Y. and J.L designed the study; Y.D., Y.B., J.W. and Z.Y. supervised the field study; J.L., Y.D. and Z.Y. acquired and analyzed the data; J.L. interpreted the data and drafted the first manuscript; and Z.Y., Y.D., C.Z. and F.J. provided critical intellectual feedback to help revise the manuscript. All authors have read and approved the final manuscript.

Availability of data and materials

This dataset is available from the corresponding author on reasonable request.

Patient consent for publication

Not required

Disclosure of prior presentation of study data as an abstract or poster

None

Acknowledgements

The authors would like to thank research teams from 12 sample sites for their hard work in the data collection. The authors also want to thank all the mothers who participated in this study.

For peer review only

Abstract

Objective: To explore and compare the predictors for exclusive breastfeeding (EBF) among migrant and non-migrant mothers in China.

Design: A large-scale cross-sectional study.

Setting: 12 counties/districts were covered in China.

Participants: A total number of 10,408 mothers were recruited, of which 3,571 mothers of 0-5 month-old-infants in urban China were used for analysis.

Outcome: The practice of EBF was calculated based on the foods and drinks consumed in the last 24-hours, as recommended by the World Health Organization.

Results: Around 30% of Chinese mothers with 0-5 month-old-infants, practiced EBF in urban areas, with no significant difference for migrant and non-migrant mothers (P=0.433). Among the migrant mothers, factors associated with EBF included residence in big cities [AOR 1.68 (1.20 - 2.34)], premature birth [AOR 0.27 (0.09 - 0.81)], knowledge on EBF [AOR 2.00 (1.51 - 2.65)], low intention of breastfeeding in the first month postpartum [AOR 0.59 (0.36 - 0.97)], and mothers working in agriculture-related fields or as casual workers [AOR 1.77 (1.18 – 2.64)]. Among non-migrant mothers, in addition to similar predictors including residence in big cities [AOR 1.40 (1.13 - 1.73)], knowledge on EBF [AOR 1.25 (1.02 - 1.53)] and low intention of breastfeeding in the first month postpartum [AOR 0.46 (0.31 - 0.70)], early initiation of breastfeeding (EIBF) [AOR 1.78 (1.35 - 2.33)] and cesarean delivery [AOR 0.74 (0.60 - 0.89)] were also factors associated with EBF.

Conclusions: There was no significant difference in the prevalence of EBF between migrant and non-migrant mothers in urban China. Premature birth and maternal occupation in agriculture-related fields or casual work were distinctive factors

For peer review only

associated with EBF for migrants, while EIBF and cesarean delivery were unique predictors for non-migrants.

Strengths and limitations of this study

- This is the first and largest study to explore and compare breastfeeding practices and its associate factors among migrant and non-migrant mothers in urban China.
- Mothers in large cities were more likely to practice EBF when compared with mothers in medium and small cities, regardless of their migrant status.
- The mothers covered in this study may not be representative of mothers in urban areas since we only invited mothers who brought their infants to immunization clinics to participate, which may overestimate the prevalence of exclusive breastfeeding.
- Since it is not a nationally representative study, the results cannot be generalized to all the urban areas of China.

For peer review only

For peer review only - http://bmjopen.bmj.com/site/about/guidelines.xhtml

Introduction

China has witnessed rapid economic growth in the past decades. Cross-country evidence showed that the prevalence of breastfeeding decreased with the increase in gross domestic product per capita[1]. Similar to the global trend, the prevalence of exclusive breastfeeding (EBF) in China dropped from 27.6% in 2008 to 20.7% in 2013[2,3]. Rapid economic development is inseparable from an increasing number of domestic migrants[4]. China has experienced an unprecedented internal migration in world history. On the one hand, better health infrastructures in cities can provide migrants with better health care services[5]. On the other hand, the household registration system and limited insurance coverage put migrants in a vulnerable situation with less potential access to urban benefits[6]. In addition to socio-economic and cultural differences between migrant and non-migrant women in China, previous studies also found that migrant women had poor knowledge and limited utilization of maternal health care services[7–9].

A large number of previous studies have focused on immigrant mothers, who moved to a country which was not their country of origin[10]. Immigrant mothers were found to be more likely to initiate and continue breastfeeding than non-immigrant mothers. However, EBF remained a challenge for both groups[10]. Little attention has been paid to breastfeeding practices and its associated factors for domestic migrant mothers. Previous literature in China disclosed the disparities in breastfeeding practices between urban and rural areas[3,11], employed and unemployed mothers[12], Han and other minority groups[13], and highly-educated and less-educated mothers[14]. Yet, few studies have examined the predictors of breastfeeding practices among migrant mothers. To fill this knowledge gap, we analyzed a large-scale cross-sectional survey

For peer review only

data to examine the similarities and disparities in EBF and its associated factors, among migrant and non-migrant mothers in China.

Methods

Study design and participants

Data for this study was derived from a large-scale cross-sectional survey in China initiated by the China Development Research Foundation in 2017. This survey was designed by an independent team at the National Institute for Nutrition and Health of the Chinese Center for Disease Control and Prevention (NINH, China CDC). Calculation of sample size was based on the expected prevalence of EBF for 0-5 month-old-infants.

We adopted a multi-stage stratified cluster sampling approach while selecting the survey sample. In the first stage, all districts/counties were categorized into 4 strata, of which 12 districts/counties were selected (4 from large cities, 4 from medium and small cities, 2 from normal rural areas and 2 from poor rural areas). The selection of districts/counties considered their geographic distributions, executive capacities, collaboration of the provincial level CDC, and their population sizes. In the second stage, four clusters were randomly selected using the probability proportional to size sampling in each selected district/county. One cluster usually had one community health center. In the last stage, mothers who brought their 0-11 month-old-infants to immunization clinics, were randomly invited to participate in the survey until the designed sample size was met. The inclusion criteria of this study were mothers of 0-11 month-old-infants who signed the informed consent form, had no psychiatric disorders and were able to answer questions clearly. To decrease the reporting bias of

For peer review only

feeding practice, we only invited mothers who were the primary caregivers of their infants. In total, 10,408 mothers of 0-11 month-old-infants were interviewed.

Patient and public involvement

This survey was undertaken by mothers of 0-11 month-old-infants in China to understand the prevalence of EBF and its associated factors. Neither these mothers nor the public were involved in the study design and implementation. The study results will be disseminated to the public through media briefings and scientific publications.

Data collection

This survey was conducted by the NINH, China CDC in collaboration with 12 teams at the provincial level CDC from September 2017 to January 2018. Data were collected through face-to-face interviews using a structured questionnaire programmed into smartphones or tablets. The questionnaire was developed by NINH, China CDC, which comprised eight sections: demographic characteristics, breastfeeding practices, maternal and child health, supportive environment for breastfeeding, health service, workplace, social environment and culture, and household financial situation related factors. The study design was approved by the Medical Ethics Committee at the NINH, China CDC. We obtained written consent forms from all mothers.

Statistical Analysis

We used the indicators recommended by the World Health Organization (WHO) to assess the feeding practice of 0-5 month-old-infants, based on the foods and drinks consumed in the last 24-hours. We defined the practice of being fed exclusively with breast milk in the last 24-hours as *exclusive breastfeeding*.

For peer review only - http://bmjopen.bmj.com/site/about/guidelines.xhtml

We first used descriptive analysis to report the selected characteristics, and then assessed their differences between migrant and non-migrant mothers, using Pearson's Chi-square test. Potential predictors were selected through reviewing relevant literature[15,16]. Chi-square tests were then used to examine the potential predictors of EBF for migrant and non-migrant mothers, respectively.

Socio-demographic characteristics included maternal age (<=25 years old, 26-35 years old and >=36 years old), maternal ethnicity (Han and others), maternal and paternal education (junior high school or less, high school, and college or higher education), maternal and paternal occupation (unemployed, agriculture-related or casual work, industry or business-related, professional or white-collar), employment type of mothers (formal and informal) and place of residence (big cities, and small and medium cities). Obstetrical history comprised parity (1 and >=2), premature birth (yes and no), low birthweight (yes and no), mode of delivery (vaginal and cesarean) and delivery hospital type (hospitals at the municipal level or above, hospitals at the district/county level and others). Breastfeeding-related factors included early initiation of breastfeeding (EIBF) (yes and no), knowledge on EBF (yes and no), low intention of breastfeeding within 1 month postpartum (yes and no) and postpartum breastfeeding difficulties (yes and no). We defined EIBF as the practice of putting infants to the breast of their mothers within the first hour of birth based on the definition of the WHO [17]. Social influence denoted whether her partner, friend, mother or mother-in-law agreed that breastmilk was better than breastmilk substitutes. Health-seeking behaviors comprised the timing of considering how to feed the infants and attending antenatal visits, breastfeeding education session or mother groups.

We then performed logistic regressions to identify predictors of EBF. The selection of covariate variables was based on the *p*-values of Chi-square tests ($P<0.05$).

For peer review only - http://bmjopen.bmj.com/site/about/guidelines.xhtml

Odds ratios and 95% confidence intervals were displayed. The data analyses were performed by Stata version 15.0 (Stata Corporation, USA). We set the level of statistical significance at α=0.05.

For peer review only

Results

Sample description and factors associated with EBF

Considering that migrants usually concentrate in urban areas, we only focused on 3,571 mothers of 0-5 month-old-infants who lived in big, medium and small cities. Migrant mothers were defined as those who had resided in a specific county for a month or more and had been in a place different from their registered county. We excluded 172 observations due to the missing values of potential predictors, which left 3,399 (>95% of the original sample) observations for statistical analysis, of which 2,199 were non-migrants and 1,200 were migrants. A sensitivity analysis using the full sample revealed very similar migrant distribution to the one shown in this study.

Table 1 displayed the summary statistics and results of the bivariate analysis, reflecting significant differences in the distribution of selected characteristics and associated factors of EBF among non-migrant and migrant mothers. The prevalence of EBF was 30.3% among non-migrant mothers, which was not statistically different from 29.0% migrant mothers (P=0.433).

Migrant mothers were statistically different from non-migrant mothers in age, ethnicity, occupation (her and her partner's), formal employment status and place of residence. More than 60% of mothers were aged 26-35 years in both groups, while the proportion of mothers above 36 years was significantly higher in non-migrant mothers (P<0.001). The likelihood of being of Han ethnicity, working as a professional or white-collar worker, and being formally employed was statistically higher in non-migrant mothers than migrant mothers as well (Table 1). Partners of non-migrant mothers were more likely to work as professionals or white-collar workers. For non-migrant mothers, socio-demographic characteristics like maternal education and occupation, paternal education, employment status of mothers and place of residence were significantly

For peer review only - http://bmjopen.bmj.com/site/about/guidelines.xhtml

associated with EBF (*P*<0.001). For migrant mothers, in addition to maternal education and occupation, paternal education, employment status of mothers, and place of residence, the ethnicity of mothers also significantly affected the prevalence of EBF. Mothers or their partners with a college or higher education were more likely to practice EBF in both groups. The prevalence of EBF was higher in mothers who had a job, were formally employed, and lived in big cities among both migrant and non-migrant mothers. Mothers from the Han ethnic group were more likely to practice EBF than mothers from minority groups among the migrant group (Table 1).

Among factors about obstetrical history, the percentage of having only one child among migrant mothers was statistically higher than for non-migrant mothers (*P*<0.001). The proportions of premature birth and low birthweight, were quite similar between migrant and non-migrant mothers, while the rate of cesarean section was statistically higher in non-migrant mothers than migrant mothers. Cesarean section was significantly associated with lower prevalence of EBF for both migrant and non-migrant mothers. However, for migrant women, premature birth and low birthweight of infants, were also negatively related to EBF. A significantly larger proportion of migrant mothers delivered their infants in hospitals at the municipal level or above (*P*<0.001). Mothers who delivered infants in hospitals at the district/county level were less likely to practice breastfeeding in both groups.

EIBF, having a low intention of breastfeeding within 1 month postpartum and breastfeeding difficulties postpartum, occurred more frequently among migrant mothers than non-migrant mothers, while only the prevalence of having breastfeeding difficulties postpartum was statistically different between the two groups. Unlike the non-migrant group where EIBF was associated with EBF significantly (*P*<0.001), migrant mothers were more likely to practice EBF if they had breastfeeding difficulties

For peer review only - http://bmjopen.bmj.com/site/about/guidelines.xhtml

postpartum. More than half of the mothers knew the definition of EBF, and around 10% of them had a low intention of breastfeeding within 1 month postpartum in both groups. Mothers in both groups were more likely to practice EBF if they knew its definition and were less likely to do it if their intentions of breastfeeding were low within 1 month postpartum.

Even though more than 80% of mothers received positive influence from their partners, friends, mothers or mothers-in-law about breastmilk being better than breastmilk substitutes, these influences differed among migrant and non-migrant mothers. The influence from partner and friends was significantly associated with EBF for non-migrant mothers, while the influence from their partners and their own mothers were significantly associated with EBF for migrant mothers.

Non-migrant mothers were more likely to adopt health-seeking behaviors like considering how to feed their infants before pregnancy and attending antenatal visits, breastfeeding education sessions, and mother groups, than migrant mothers. Only the timing of considering how to feed their infants was statistically different between the two groups. Migrant mothers who considered how to feed their infants before pregnancy were more likely to practice EBF. Attending breastfeeding education sessions significantly affected the EBF practice for non-migrant mothers only.

For peer review only

Table 1 Factors associated with EBF practice among mothers of 0-5 month-old-infants by migration status

	Non-migrant mothers (n=2199)					Migrant mothers (n=1200)					
	Total		EBF			Total		EBF			
	N	%	n	%	*P*-value[b]	N	%	n	%	*P*-value[b]	*P*-value[a]
Socio-Demographic characteristics											
Maternal age					0.740					0.536	<0.001
<=25 years old	448	20.4	129	28.8		283	23.6	83	29.3		
26-35 years old	1411	64.2	432	30.6		799	66.6	236	29.5		
36+ years old	340	15.5	105	30.9		118	9.8	29	24.6		
Maternal ethnicity					0.080					0.019	0.002
Others	360	16.4	123	34.2		248	20.7	57	23.0		
Han	1839	83.6	543	29.5		952	79.3	291	30.6		
Maternal education					<0.001					<0.001	0.489
Junior high school or less	666	30.3	167	25.1		361	30.1	79	21.9		
High school	422	19.2	106	25.1		212	17.7	52	24.5		
College or higher education	1111	50.5	393	35.4		627	52.3	217	34.6		
Maternal occupation					<0.001					0.001	<0.001
Unemployed	776	35.3	201	25.9		449	37.4	101	22.5		
Agriculture-related or casual work	370	16.8	101	27.3		211	17.6	70	33.2		
Industry or business-related	325	14.8	106	32.6		237	19.8	82	34.6		
Professionals or white-collars	728	33.1	258	35.4		303	25.3	95	31.4		
Paternal education					<0.001					<0.001	0.211
Junior high school or less	696	31.7	174	25.0		345	28.8	80	23.2		
High school	404	18.4	90	22.3		233	19.4	54	23.2		
College or higher education	1099	50.0	402	36.6		622	51.8	214	34.4		
Paternal occupation					0.434					0.473	<0.001
Unemployed	112	5.1	37	33.0		49	4.1	11	22.4		
Agriculture-related or casual work	597	27.1	169	28.3		317	26.4	101	31.9		
Industry or business-related	660	30.0	195	29.5		453	37.8	127	28.0		
Professionals or white-collars	830	37.7	265	31.9		381	31.8	109	28.6		
Mother was formally employed					<0.001					<0.001	0.021
No	1356	61.7	365	26.9		788	65.7	197	25.0		
Yes	843	38.3	301	35.7		412	34.3	151	36.7		
Place of residence					<0.001					<0.001	<0.001

For peer review only - http://bmjopen.bmj.com/site/about/guidelines.xhtml
For peer review only

Small and medium sized cities	1222	55.6	299	24.5		411	34.3	82	20.0		
Big cities	977	44.4	367	37.6		789	65.8	266	33.7		
Obstetrical history											
Parity					0.875					0.105	<0.001
>=2	1181	53.7	356	30.1		509	42.4	135	26.5		
1	1018	46.3	310	30.5		691	57.6	213	30.8		
Premature birth					0.877					0.006	0.430
No	2112	96.0	639	30.3		1159	96.6	344	29.7		
Yes	87	4.0	27	31.0		41	3.4	4	9.8		
Low birthweight					0.389					0.019	0.807
No	2104	95.7	641	30.5		1146	95.5	340	29.7		
Yes	95	4.3	25	26.3		54	4.5	8	14.8		
Mode of delivery					<0.001					0.016	0.010
Vaginal	1329	60.4	438	33.0		779	64.9	244	31.3		
Cesarean	870	39.6	228	26.2		421	35.1	104	24.7		
Delivery hospital type					0.103					0.095	<0.001
Hospitals at the municipal or above level	1348	61.3	423	31.4		849	70.8	259	30.5		
Hospitals at the district/county level	796	36.2	222	27.9		316	26.3	77	24.4		
Others	55	2.5	21	38.2		35	2.9	12	34.3		
Breastfeeding related factors											
Early initiation of breastfeeding					<0.001					0.098	0.216
No	1917	87.2	538	28.1		1028	85.7	289	28.1		
Yes	282	12.8	128	45.4		172	14.3	59	34.3		
Knowledge on EBF					<0.001					<0.001	0.708
No	940	42.7	238	25.3		505	42.1	98	19.4		
Yes	1259	57.3	428	34.0		695	57.9	250	36.0		
Low intention of breastfeeding within 1 month postpartum					<0.001					0.029	0.115
No	2018	91.8	635	31.5		1082	90.2	324	29.9		
Yes	181	8.2	31	17.1		118	9.8	24	20.3		
Breastfeeding difficulties postpartum					0.436					<0.001	0.022
No	1567	71.3	467	29.8		810	67.5	210	25.9		
Yes	632	28.7	199	31.5		390	32.5	138	35.4		
Social influence											
Partner supports breastfeeding					0.012					0.031	0.868

For peer review only - http://bmjopen.bmj.com/site/about/guidelines.xhtml

No	270	12.3	64	23.7		145	12.1	31	21.4		
Yes	1929	87.7	602	31.2		1055	87.9	317	30.0		
Friend supports breastfeeding					0.031					0.217	0.719
No	318	14.5	80	25.2		179	14.9	45	25.1		
Yes	1881	85.5	586	31.2		1021	85.1	303	29.7		
Mother supports breastfeeding					0.295					0.014	0.259
No	255	11.6	70	27.5		155	12.9	32	20.6		
Yes	1944	88.4	596	30.7		1045	87.1	316	30.2		
Mother-in-law supports breastfeeding					0.740					0.198	0.114
No	312	14.2	92	29.5		147	12.3	36	24.5		
Yes	1887	85.8	574	30.4		1053	87.8	312	29.6		
Health-seeking behaviors											
Timing of considering how to feed the infants					0.490					0.002	<0.001
Before pregnancy	1208	54.9	365	30.2		532	44.3	178	33.5		
During pregnancy	646	29.4	188	29.1		429	35.8	119	27.7		
After birth	345	15.7	113	32.8		239	19.9	51	21.3		
Attend Antenatal visits					0.992					0.243	0.067
No	89	4.0	27	30.3		65	5.4	23	35.4		
Yes	2110	96.0	639	30.3		1135	94.6	325	28.6		
Attend breastfeeding education sessions					0.046					0.249	0.540
No	1101	50.1	312	28.3		614	51.2	169	27.5		
Yes	1098	49.9	354	32.2		586	48.8	179	30.5		
Attend mother groups					0.082					0.999	0.342
No	1373	62.4	434	31.6		769	64.1	223	29.0		
Yes	826	37.6	232	28.1		431	35.9	125	29.0		

[a] *P*-value of chi-square test to compare distribution of covariate variables between migrant and non-migrant mothers.
[b] *P*-value of chi-square test to identify risk factors of exclusive breastfeeding practice among migrant and non-migrant mothers respectively.

For peer review only

Predictors of breastfeeding practice among migrant and non-migrant mothers

We then performed logistic regression to determine the predictors of EBF, respectively. We only kept covariates with *p*-values smaller than 0.05 in Table 1.

Among socio-demographic factors, place of residence was significantly associated with EBF. Migrants living in big cities were more likely to practice EBF than those in small or medium cities (adjusted OR(AOR) =1.68, 95% CI 1.20 - 2.34) after adjusting for other potential confounders. The odds of EBF were lower among migrant mothers with preterm infants (AOR= 0.27, 95% CI 0.09 - 0.81) and having a low intention of breastfeeding in the first month postpartum (AOR= 0.59, 95% CI 0.36 - 0.97). Migrant mothers who worked in agriculture-related fields or as casual workers (AOR=1.77, 95% CI 1.18 - 2.64), and who knew EBF (AOR= 2.00, 95% CI 1.51 - 2.65) were more likely to practice EBF (Table 2).

Among non-migrant mothers, we found that residence in big cities (AOR=1.40, 95% CI 1.13 - 1.73), practicing EIBF (AOR=1.78, 95% CI 1.35 - 2.33), and knowledge on EBF (AOR=1.25, 95% CI 1.02 - 1.53), were significantly associated with optimal breastfeeding practice. However, cesarean section (AOR=0.74, 95% CI 0.60 - 0.89) and low intention of breastfeeding (AOR=0.46, 95% CI 0.31 - 0.70) significantly decreased the likelihood to practice EBF (Table 3).

For peer review only - http://bmjopen.bmj.com/site/about/guidelines.xhtml

Table 2 Predictors of EBF practice among migrant mothers of 0-5 month-old-infants (n=1200)

Risk factors	AOR	95% CI
Maternal ethnicity		
Others		
Han	1.14	(0.79 - 1.65)
Maternal education		
Junior high school or less		
High school	1.15	(0.72 - 1.83)
College or higher education	1.38	(0.83 - 2.28)
Maternal occupation		
Unemployed		
Agriculture-related or casual work	1.77**	(1.18 - 2.64)
Industry or business related	1.25	(0.84 - 1.86)
Professionals or white-collars	0.98	(0.61 - 1.55)
Paternal education		
Junior high school or less		
High school	0.71	(0.45 - 1.13)
College or higher education	0.90	(0.55 - 1.47)
Mather was formally employed		
No		
Yes	1.29	(0.88 - 1.91)
Place of residence		
Small and medium cities		
Big cities	1.68**	(1.20 - 2.34)
Premature birth		
No		
Yes	0.27*	(0.09 - 0.81)
Low birthweight		
No		
Yes	0.66	(0.29 - 1.52)
Mode of delivery		
Vaginal		
Cesarean	0.86	(0.65 - 1.14)
Knowledge on EBF		
No		
Yes	2.00***	(1.51 - 2.65)
Low intention of breastfeeding within 1 month postpartum		
No		
Yes	0.59*	(0.36 - 0.97)
Breastfeeding difficulties postpartum		
No		
Yes	1.32	(0.99 - 1.75)
Partner supports breastfeeding		
No		
Yes	1.34	(0.77 - 2.35)
Grandmother supports breastfeeding		
No		
Yes	1.51	(0.88 - 2.60)
Timing of considering how to feed the infants		
Before pregnancy		
During pregnancy	0.87	(0.65 - 1.17)
After birth	0.75	(0.51 - 1.11)

Odds ratios were adjusted for all the covariates in the model. * $P < 0.05$, ** $P < 0.01$, *** $P < 0.001$.

For peer review only

Table 3 Predictors of EBF practice among non-migrant mothers of 0-5 month-old-infants (n=2199)

Risk factors	AOR	95% CI
Maternal education		
Junior high school or less		
High school	0.98	(0.70 - 1.36)
College or higher education	0.98	(0.66 - 1.45)
Maternal occupation		
Unemployed		
Agriculture-related or casual work	1.15	(0.85 - 1.54)
Industry or business related	1.15	(0.84 - 1.59)
Professionals or white-collar	1.20	(0.85 - 1.70)
Paternal education		
Junior high school or less		
High school	0.81	(0.58 - 1.13)
College or higher education	1.33	(0.92 - 1.93)
Mather was formally employed		
No		
Yes	0.98	(0.72 - 1.35)
Place of residence		
Small and medium cities		
Big cities	1.40**	(1.13 - 1.73)
Mode of delivery		
Vaginal		
Cesarean	0.74**	(0.60 - 0.89)
Early initiation of breastfeeding		
No		
Yes	1.78***	(1.35 - 2.33)
Knowledge on EBF		
No		
Yes	1.25*	(1.02 - 1.53)
Low intention of breastfeeding within 1 month postpartum		
No		
Yes	0.46***	(0.31 - 0.70)
Partner supports breastfeeding		
No		
Yes	1.22	(0.84 - 1.78)
Friend supports breastfeeding		
No		
Yes	1.14	(0.81 - 1.61)
Attend breastfeeding education sessions		
No		
Yes	1.11	(0.92 - 1.36)

Odds ratios were adjusted for all the covariates in the model. * $P < 0.05$, ** $P < 0.01$, *** $P < 0.001$.

For peer review only

For peer review only - http://bmjopen.bmj.com/site/about/guidelines.xhtml

Discussion

In this study, we found that breastfeeding practice was suboptimal in both migrant and non-migrant mothers. The prevalence of EBF among non-migrant women was not significantly different from that of migrant mothers. This is the first and largest study to explore and compare breastfeeding practices and their potential predictors among migrant and non-migrant mothers in China. Multivariate regression results suggested that similar predictors of EBF for mothers in both groups included residence in big cities, knowledge on EBF, and low intention of breastfeeding in the first month postpartum. Mothers in big cities were approximately 1.5 times more likely to practice EBF. Among migrant mothers, having premature infants and working in agriculture-related fields or as casual workers, were associated with EBF practice. However, for non-migrant mothers, EIBF and cesarean delivery were unique predictors of EBF practice.

Living in big cities was the only common variable in demographic characteristics that were significantly associated with EBF practice in both the groups. Previous literature also suggested a higher rate of EBF in big cities than in middle and small cities in China using nationally representative data derived from the Chinese National Nutrition and Health Survey conducted in 2013[3]. This is possibly due to better knowledge and supportive environment for mothers in big cities than those living in small and medium cities regardless of their migration status. For example, the proportion of mothers who received information on encouraging breastfeeding was higher in big cities (55.3%) than in small and medium cities (44.7%), and more than 92% of them received encouragement from the delivery hospital in big cities. This revealed that both migrant and non-migrant mothers may benefit from better breastfeeding-related education and support, in big cities in China.

Another common predictor of EBF for both migrant and non-migrant mothers was knowledge on EBF. Some of the previous studies used knowledge scores on breastfeeding benefits as a predictor of breastfeeding practice, but they failed to provide consistent evidence

For peer review only - http://bmjopen.bmj.com/site/about/guidelines.xhtml

on its association with EBF[11,18]. However, we only focused on the key message that infants should be exclusively breastfed for the first 6 months. This implies that future education intervention projects should pay more attention to disseminating core messages in promoting optimal breastfeeding practices.

Additionally, we also found that having a low intention of breastfeeding in the first month postpartum significantly decreased the odds of practicing EBF for both groups. Cracked nipples or nipple pain, and insufficient breastmilk supply ranked as the top two reasons, which together accounted for around half of all the answers causing low breastfeeding intention. In alignment with previous studies, nipple pain or fissure within the first month postpartum was the commonest reason for the low intention of breastfeeding among mothers regardless of their migration status[19,20]. Given that nipple pain was often attributed to incorrect positioning and attachment[20], health care providers need to find effective measures to help establish proper positioning for mothers, good attachment of the babies to the breast as well as effective sucking. In addition, insufficient milk supply was frequently reported by previous studies as the leading cause of breastfeeding cessation or its exclusivity[21,22]. However there is limited evidence on how to design interventions to address maternal perceptions or concerns of insufficient milk supply[23,24]. Further studies are needed to identify effective interventions regarding the maternal perception of milk supply.

In addition to the common predictors of EBF, as explained above, there were unique risk factors for migrant and non-migrant mothers, respectively. Among migrant mothers, having premature infants significantly decreased the prevalence of EBF. The EBF rate for migrant mothers with preterm infants was 9.8%, which was vastly lower than that of non-migrant mothers (31.0%). The low EBF rate could be attributable to mother-infant separation in neonatal intensive care units (NICUs) in China[25], which resulted in many difficulties and challenges for mothers with premature infants. The disparity in the EBF rate of premature

For peer review only

infants may come from the different choices of hospitalization services between migrant and non-migrant mothers. Even though migrant mothers are more likely to deliver their infants in higher level hospitals in general, the proportion of migrant mothers with premature infants who delivered in hospitals at the municipal or higher levels was 65.9%, lower than that of non-migrant mothers (73.6%). In China, high-level hospitals are believed to have well-trained health workers and high-quality medical care [26]. An internationally well-known practice such as Kangaroo Care (KC), has only been implemented in some NICUs of high-level hospitals in China as pilot studies, for promoting the development of premature infants [27]. Implementation of KC was hindered by the limits on parental visitation, inconsistent guidelines and safety fears of the medical staff [27]. Medical practice in NICUs may need to encourage more involvement of parents to care for their infants.

Additionally, migrant mothers working in agriculture-related fields or as casual workers, were more likely to practice EBF. This is possibly because when compared with mothers working in other fields, they have more flexible time, and the shortest commuting time of 29-minutes for those who have already gone backed to work. When compared with unemployed mothers, these migrant mothers were more likely to adopt health seeking behaviors like starting to consider how to feed their infants before pregnancy and attending breastfeeding education sessions in hospitals. These findings were in alignment with previous studies, finding that mothers who worked full-time were less likely to initiate or continue breastfeeding[28]. Our results suggested that working was not necessarily a barrier for breastfeeding, allowing lactating mothers to work with a flexible schedule that may provide support for breastfeeding.

Among non-migrant mothers, EIBF and cesarean section were distinctive predictors of EBF. The rate of EIBF was 12.8% in non-migrant mothers, which was a little higher than the 9% found in a study in the Sichuan province[29], but much lower than the national

For peer review only - http://bmjopen.bmj.com/site/about/guidelines.xhtml

prevalence of 28.7% in 2013[30] and the 59.4% found in a study in central and western China[31]. A large regional disparity of EIBF in China may be due to the delayed process of implementing Early Essential Newborn Care (EENC) in China. Even in places where EENC had been implemented, skin-to-skin contact was often interrupted due to inadequate facilities and early initiation of breastfeeding was difficult to be ensured[32]. Hospitals in China need to speed up their process of adopting EENC recommended practices and national policies are needed to be established to help hospitals and health workers overcome obstacles in implementing these practices.

For obstetrical history-related factors, we also found that the prevalence of cesarean delivery was high and negatively associated with EBF among non-migrant mothers only. The prevalence of cesarean delivery was 39.6% among non-migrant mothers, which was higher than the national average of 36.7% in 2018[33]. Even though China had made efforts in decreasing cesarean delivery in the past decade[34], the national prevalence is still far higher than the suggested prevalence of 10-15% by the WHO[35]. The government and health facilities need to make effective policies and measures in addressing non-clinical reasons for preferring a cesarean delivery, such as maternal request and perceived convenience[36].

This study derived data from a large-scale cross-sectional survey covering 8 sample sites in urban areas, which is the first and largest study to explore and compare breastfeeding practices and their potential predictors among migrant and non-migrant mothers in China. Findings in this study filled the knowledge gap in the similarities and differences in the predictors of EBF among migrant and non-migrant mothers, which had important implications for future studies as well as health interventions to promote optimal breastfeeding practices in China.

However, our study still faced the following limitations. First, differences in the predictors of EBF among migrant and non-migrant mothers revealed in this study, may come

For peer review only - http://bmjopen.bmj.com/site/about/guidelines.xhtml

from different choices in hospitalization services. Migrant mothers may have restricted access to better health services due to social exclusions, lack of social networks, and health insurance. Future studies need to take a closer look at the differences in health facility choices among migrant and non-migrant mothers and identify potential obstacles faced by the migrants. Second, mothers covered in this study may not be representative of the mothers in general. That is because we only interviewed mothers who brought their infants into immunization clinics at the time of the interview, which naturally excluded children who were left behind in their early childhood and may overestimate the prevalence of EBF for both groups [37,38]. A low rate of EBF would be really problematic since it will not only result in nutrition-related harm to the early childhood development of children, but also increase the health risks of mothers in China[1]. Third, when selecting sample sites in the first stage, we considered the executive capacities, and collaboration of the provincial level CDC. Thus, sample sites selected in this study to some extent reflected a higher efficiency of health systems at the county/district level. Caution is needed when interpreting these results and the results disclosed in this study cannot be generalized to the whole urban areas of China.

To conclude, this study found that there was no significant difference in the prevalence of EBF between migrant and non-migrant mothers in our sampled urban areas of China. Even though the predictors of EBF shared some similarities between migrant and non-migrant mothers, future interventions may still need to adopt different strategies in promoting optimal breastfeeding practices in different groups.

For peer review only - http://bmjopen.bmj.com/site/about/guidelines.xhtml

Reference

1 Victora CG, Bahl R, Barros AJD, *et al.* Breastfeeding in the 21st century: epidemiology, mechanisms, and lifelong effect. *Lancet* 2016;**387**:475–90. doi:10.1016/S0140-6736(15)01024-7

2 Center for Health Statistics and Information at Ministry of Health of People's Republic of China. *An Analysis Report of National Health Services Survey in China,2008*. Beijing: 2009.

3 Duan Y, Yang Z, Lai J, *et al.* Exclusive Breastfeeding Rate and Complementary Feeding Indicators in China: A National Representative Survey in 2013. *Nutrients* 2018;**10**:249. doi:10.3390/nu10020249

4 Wang X-R, Hui EC-M, Sun J-X. Population migration, urbanization and housing prices: Evidence from the cities in China. *Habitat Int* 2017;**66**:49–56. doi:10.1016/j.habitatint.2017.05.010

5 Song Y, Sun W. Health Consequences of Rural-to-Urban Migration: Evidence from Panel Data in China. *Health Econ* 2016;**25**:1252–67. doi:10.1002/hec.3212

6 Peng Y, Chang W, Zhou H, *et al.* Factors associated with health-seeking behavior among migrant workers in Beijing, China. *BMC Health Serv Res* 2010;**10**:69. doi:10.1186/1472-6963-10-69

7 Zhao Q, Kulane A, Gao Y, *et al.* Knowledge and attitude on maternal health care among rural-to-urban migrant women in Shanghai, China. *BMC Womens Health* Published Online First: 2009. doi:10.1186/1472-6874-9-5

8 Zhao Q, Huang ZJ, Yang S, *et al.* The utilization of antenatal care among rural-to-urban migrant women in Shanghai: A hospital-based cross-sectional study. *BMC Public Health* Published Online First: 2012. doi:10.1186/1471-2458-12-

For peer review only - http://bmjopen.bmj.com/site/about/guidelines.xhtml

1012

9 WEI X, PEARSON S, ZHANG Z, *et al.* COMPARING KNOWLEDGE AND USE OF HEALTH SERVICES OF MIGRANTS FROM RURAL AND URBAN AREAS IN KUNMING CITY, CHINA. *J Biosoc Sci* 2010;**42**:743–56. doi:10.1017/S0021932010000313

10 Dennis C, Shiri R, Brown HK, *et al.* Breastfeeding rates in immigrant and non-immigrant women: A systematic review and meta-analysis. *Matern Child Nutr* 2019;**15**:e12809. doi:10.1111/mcn.12809

11 Gao H, Wang Q, Hormann E, *et al.* Breastfeeding practices on postnatal wards in urban and rural areas of the Deyang region, Sichuan province of China. *Int Breastfeed J* 2016;**11**:11. doi:10.1186/s13006-016-0070-0

12 Li L, Li S, Ali M, *et al.* Feeding practice of infants and their correlates in urban areas of Beijing, China. *Pediatr Int* 2003;**45**:400–6. doi:10.1046/j.1442-200X.2003.01754.x

13 Xu F, Binns C, Nazi G, *et al.* A comparison of breastfeeding among Han, Uygur and other ethnic groups in Xinjiang, PR China. *BMC Public Health* 2006;**6**:196. doi:10.1186/1471-2458-6-196

14 Zhao J, Zhao Y, Du M, *et al.* Maternal education and breastfeeding practices in China: A systematic review and meta-analysis. *Midwifery* 2017;**50**:62–71. doi:10.1016/j.midw.2017.03.011

15 Rollins NC, Bhandari N, Hajeebhoy N, *et al.* Why invest, and what it will take to improve breastfeeding practices? *Lancet* 2016;**387**:491–504. doi:10.1016/S0140-6736(15)01044-2

16 Dennis C-L, Gagnon A, Van Hulst A, *et al.* Prediction of Duration of Breastfeeding among Migrant and Canadian-Born Women: Results from a

For peer review only

For peer review only - http://bmjopen.bmj.com/site/about/guidelines.xhtml

Multi-Center Study. *J Pediatr* 2013;**162**:72–9. doi:10.1016/j.jpeds.2012.06.041

17 Daelmans B, Dewey K, Arimond M. New and Updated Indicators for Assessing Infant and Young Child Feeding. *Food Nutr Bull* 2009;**30**:S256–62. doi:10.1177/15648265090302S210

18 Ling Shi, Jingxu Zhang, Yan Wang, *et al.* Breastfeeding in Rural China: Association Between Knowledge, Attitudes, and Practices. *J Hum Lact* 2008;**24**:377–85. doi:10.1177/0890334408319157

19 McCann MF, Baydar N, Williams RL. Breastfeeding Attitudes and Reported Problems in a National Sample of WIC Participants. *J Hum Lact* 2007;**23**:314–24. doi:10.1177/0890334407307882

20 Kent J, Ashton E, Hardwick C, *et al.* Nipple Pain in Breastfeeding Mothers: Incidence, Causes and Treatments. *Int J Environ Res Public Health* 2015;**12**:12247–63. doi:10.3390/ijerph121012247

21 Balogun OO, Dagvadorj A, Anigo KM, *et al.* Factors influencing breastfeeding exclusivity during the first 6 months of life in developing countries: a quantitative and qualitative systematic review. *Matern Child Nutr* 2015;**11**:433–51. doi:10.1111/mcn.12180

22 Flaherman VJ, Beiler JS, Cabana MD, *et al.* Relationship of newborn weight loss to milk supply concern and anxiety: the impact on breastfeeding duration. *Matern Child Nutr* 2016;**12**:463–72. doi:10.1111/mcn.12171

23 Galipeau R, Baillot A, Trottier A, *et al.* Effectiveness of interventions on breastfeeding self-efficacy and perceived insufficient milk supply: A systematic review and meta-analysis. *Matern Child Nutr* 2018;**14**. doi:10.1111/mcn.12607

24 Wood NK, Sanders EA, Lewis FM, *et al.* Pilot test of a home-based program to

For peer review only

For peer review only - http://bmjopen.bmj.com/site/about/guidelines.xhtml

prevent perceived insufficient milk. *Women and Birth* 2017;**30**:472–80. doi:10.1016/j.wombi.2017.04.006

25 Wang Y, Briere C-E, Xu W, *et al.* Factors Affecting Breastfeeding Outcomes at Six Months in Preterm Infants. *J Hum Lact* 2019;**35**:80–9. doi:10.1177/0890334418771307

26 Wang H, Zhang D, Hou Z, *et al.* Association between social health insurance and choice of hospitals among internal migrants in China: a national cross-sectional study. *BMJ Open* 2018;**8**:e018440. doi:10.1136/bmjopen-2017-018440

27 Zhang Y, Deng Q, Zhu B, *et al.* Neonatal intensive care nurses' knowledge and beliefs regarding kangaroo care in China: a national survey. *BMJ Open* 2018;**8**:e021740. doi:10.1136/bmjopen-2018-021740

28 Mandal B, Roe BE, Fein SB. The differential effects of full-time and part-time work status on breastfeeding. *Health Policy (New York)* 2010;**97**:79–86. doi:10.1016/j.healthpol.2010.03.006

29 Tang L, Binns CW, Lee AH, *et al.* Low Prevalence of Breastfeeding Initiation within the First Hour of Life in a Rural Area of Sichuan Province, China. *Birth* 2013;**40**:134–42. doi:10.1111/birt.12038

30 UNICEF. Children in China: An Atlas of Social Indicators of Children in China 2018. Beijing: 2018.

31 Guo S, Fu X, Scherpbier RW, *et al.* Breastfeeding rates in central and western China in 2010: implications for child and population health. *Bull World Health Organ* 2013;**91**:322–31. doi:10.2471/BLT.12.111310

32 Qu W, Yue Q, Wang Y, *et al.* Implementation of the Early Essential Newborn Care (EENC) on neonatal outcomes in west China: an observational study.

For peer review only

Lancet 2018;**392**:S54. doi:10.1016/S0140-6736(18)32683-7

33 Li H, Hellerstein S, Zhou Y, *et al.* Trends in Cesarean Delivery Rates in China, 2008-2018. *JAMA* 2020;**323**:89. doi:10.1001/jama.2019.17595

34 Liang J, Mu Y, Li X, *et al.* Relaxation of the one child policy and trends in caesarean section rates and birth outcomes in China between 2012 and 2016: observational study of nearly seven million health facility births. *BMJ* 2018;:k817. doi:10.1136/bmj.k817

35 Betran A, Torloni M, Zhang J, *et al.* WHO Statement on Caesarean Section Rates. *BJOG An Int J Obstet Gynaecol* 2016;**123**:667–70. doi:10.1111/1471-0528.13526

36 Long Q, Kingdon C, Yang F, *et al.* Prevalence of and reasons for women's, family members', and health professionals' preferences for cesarean section in China: A mixed-methods systematic review. *PLOS Med* 2018;**15**:e1002672. doi:10.1371/journal.pmed.1002672

37 Tian X, Ding C, Shen C, *et al.* Does Parental Migration Have Negative Impact on the Growth of Left-Behind Children?—New Evidence from Longitudinal Data in Rural China. *Int J Environ Res Public Health* 2017;**14**:1308. doi:10.3390/ijerph14111308

38 Zhang N, Bécares L, Chandola T. Does the timing of parental migration matter for child growth? A life course study on left-behind children in rural China. *BMC Public Health* 2015;**15**:966. doi:10.1186/s12889-015-2296-y

For peer review only

For peer review only - http://bmjopen.bmj.com/site/about/guidelines.xhtml

STROBE Statement—Checklist of items that should be included in reports of ***cross-sectional studies***

	Item No	Page number in manuscript	Recommendation
**Title and abstract**	1	Page 3	(*a*) Indicate the study's design with a commonly used term in the title or the abstract
		Page 3	(*b*) Provide in the abstract an informative and balanced summary of what was done and what was found
**Introduction**			
Background/rationale	2	Page 5	Explain the scientific background and rationale for the investigation being reported
Objectives	3	Page 5	State specific objectives, including any prespecified hypotheses
**Methods**			
Study design	4	Page 6	Present key elements of study design early in the paper
Setting	5	Page 6-7	Describe the setting, locations, and relevant dates, including periods of recruitment, exposure, follow-up, and data collection
Participants	6	Page 6-7	(*a*) Give the eligibility criteria, and the sources and methods of selection of participants
Variables	7	Page 7	Clearly define all outcomes, exposures, predictors, potential confounders, and effect modifiers. Give diagnostic criteria, if applicable
Data sources/ measurement	8*	Page 7	For each variable of interest, give sources of data and details of methods of assessment (measurement). Describe comparability of assessment methods if there is more than one group
Bias	9	Page 6	Describe any efforts to address potential sources of bias
Study size	10	Page 6-7	Explain how the study size was arrived at
Quantitative variables	11	Page 7-8	Explain how quantitative variables were handled in the analyses. If applicable, describe which groupings were chosen and why
Statistical methods	12	Page 7-9	(*a*) Describe all statistical methods, including those used to control for confounding
		Page 7-9	(*b*) Describe any methods used to examine subgroups and interactions
		Page 10	(*c*) Explain how missing data were addressed
		Not applicable	(*d*) If applicable, describe analytical methods taking account of sampling strategy
		Page 10	(*e*) Describe any sensitivity analyses
**Results**			
Participants	13*	Page 10	(a) Report numbers of individuals at each stage of study—eg numbers potentially eligible, examined for eligibility, confirmed eligible, included in the study, completing follow-up, and analysed
		Not applicable	(b) Give reasons for non-participation at each stage
		Not applicable	(c) Consider use of a flow diagram
Descriptive data	14*	Page 10	(a) Give characteristics of study participants (eg demographic, clinical, social) and information on exposures and potential confounders
		Not applicable	(b) Indicate number of participants with missing data for each variable of interest

For peer review only - http://bmjopen.bmj.com/site/about/guidelines.xhtml

Outcome data	15*	Page 10	Report numbers of outcome events or summary measures
Main results	16	Page 10-18	(*a*) Give unadjusted estimates and, if applicable, confounder-adjusted estimates and their precision (eg, 95% confidence interval). Make clear which confounders were adjusted for and why they were included
		Not applicable	(*b*) Report category boundaries when continuous variables were categorized
		Not applicable	(*c*) If relevant, consider translating estimates of relative risk into absolute risk for a meaningful time period
Other analyses	17	Page 16-18	Report other analyses done—eg analyses of subgroups and interactions, and sensitivity analyses
**Discussion**			
Key results	18	Page 19	Summarise key results with reference to study objectives
Limitations	19	Page 22-23	Discuss limitations of the study, taking into account sources of potential bias or imprecision. Discuss both direction and magnitude of any potential bias
Interpretation	20	Page 19-22	Give a cautious overall interpretation of results considering objectives, limitations, multiplicity of analyses, results from similar studies, and other relevant evidence
Generalisability	21	Page 22-23	Discuss the generalisability (external validity) of the study results
**Other information**			
Funding	22	Title page	Give the source of funding and the role of the funders for the present study and, if applicable, for the original study on which the present article is based

*Give information separately for exposed and unexposed groups.

Note: An Explanation and Elaboration article discusses each checklist item and gives methodological background and published examples of transparent reporting. The STROBE checklist is best used in conjunction with this article (freely available on the Web sites of PLoS Medicine at http://www.plosmedicine.org/, Annals of Internal Medicine at http://www.annals.org/, and Epidemiology at http://www.epidem.com/). Information on the STROBE Initiative is available at www.strobe-statement.org.

For peer review only

Predictors of exclusive breastfeeding practice among migrant and non-migrant mothers in urban China: results from a cross-sectional survey

Journal:	*BMJ Open*
Manuscript ID	bmjopen-2020-038268.R2
Article Type:	Original research
Date Submitted by the Author:	28-Jun-2020

Complete List of Authors:	Li, Jia; Tsinghua University School of Social Sciences, ; China Development Research Foundation,<br>Duan, Yifan; Chinese Center for Disease Control and Prevention National Institute for Nutrition and Health<br>Bi, Ye; Chinese Center for Disease Control and Prevention National Institute for Nutrition and Health<br>Wang, Jie; Chinese Center for Disease Control and Prevention National Institute for Nutrition and Health<br>Lai, Jianqiang; Chinese Center for Disease Control and Prevention National Institute for Nutrition and Health<br>Zhao, Chen; China Development Research Foundation<br>Fang, Jin; China Development Research Foundation<br>Yang, Zhenyu; Chinese Center for Disease Control and Prevention National Institute for Nutrition and Health

**Primary Subject Heading**:	Public health
Secondary Subject Heading:	Epidemiology
Keywords:	NUTRITION & DIETETICS, PUBLIC HEALTH, EPIDEMIOLOGY

SCHOLARONE™
Manuscripts

I, the Submitting Author has the right to grant and does grant on behalf of all authors of the Work (as defined in the below author licence), an exclusive licence and/or a non-exclusive licence for contributions from authors who are: i) UK Crown employees; ii) where BMJ has agreed a CC-BY licence shall apply, and/or iii) in accordance with the terms applicable for US Federal Government officers or employees acting as part of their official duties; on a worldwide, perpetual, irrevocable, royalty-free basis to BMJ Publishing Group Ltd ("BMJ") its licensees and where the relevant Journal is co-owned by BMJ to the co-owners of the Journal, to publish the Work in this journal and any other BMJ products and to exploit all rights, as set out in our licence.

The Submitting Author accepts and understands that any supply made under these terms is made by BMJ to the Submitting Author unless you are acting as an employee on behalf of your employer or a postgraduate student of an affiliated institution which is paying any applicable article publishing charge ("APC") for Open Access articles. Where the Submitting Author wishes to make the Work available on an Open Access basis (and intends to pay the relevant APC), the terms of reuse of such Open Access shall be governed by a Creative Commons licence – details of these licences and which Creative Commons licence will apply to this Work are set out in our licence referred to above.

Other than as permitted in any relevant BMJ Author's Self Archiving Policies, I confirm this Work has not been accepted for publication elsewhere, is not being considered for publication elsewhere and does not duplicate material already published. I confirm all authors consent to publication of this Work and authorise the granting of this licence.

For peer review only

Predictors of exclusive breastfeeding practice among migrant and non-migrant mothers in urban China: results from a cross-sectional survey

Running title: Exclusive breastfeeding in urban China

Jia Li, PhD[1,2], Yifan Duan, MSc[3], Ye Bi, MSc[3], Jie Wang, PhD[3], Jianqiang Lai, PhD[3], Chen Zhao, MSocsc[2], Jin Fang, PhD[2], Zhenyu Yang, PhD[3,*]

[1] Institute of Economics, School of Social Sciences, Tsinghua University, Beijing, China

[2] China Development Research Foundation, Beijing, China

[3] National Institute for Nutrition and Health, Chinese Center for Disease Control and Prevention, Beijing, China

[*] Correspondence and reprint request to: Dr Zhenyu Yang (yangzy@ninh.chinacdc.cn), National Institute for Nutrition and Health, Chinese Center for Disease Control and Prevention, No.27 Nanwei Road, Xicheng District, Beijing, China, 100050, Phone +86-10-66237198, Fax +86-10-67791992

For peer review only

**Manuscript statistics:**	Number of tables: 3
Main text word count: 3768	Number of figures: 0
Abstract word count: 277	Number of references: 46

Disclaimers

The views expressed in the article are those of the authors and do not necessarily reflect the views of the institution and funder.

For peer review only - http://bmjopen.bmj.com/site/about/guidelines.xhtml

Funding source

The study was funded by the Bill & Melinda Gates Foundation to China Development Research Foundation (Grant Number OPP1152715).

Competing interests

None of the authors had a conflict of interest related to any part of this study or manuscript.

Authors' contributions

J.L., Y.D., Z.Y. and J.L designed the study; Y.D., Y.B., J.W. and Z.Y. supervised the field study; J.L., Y.D. and Z.Y. acquired and analyzed the data; J.L. interpreted the data and drafted the first manuscript; and Z.Y., Y.D., C.Z. and F.J. provided critical intellectual feedback to help revise the manuscript. All authors have read and approved the final manuscript.

Availability of data and materials

This dataset is available from the corresponding author on reasonable request.

Patient consent for publication

Not required

Disclosure of prior presentation of study data as an abstract or poster

None

Acknowledgements

The authors would like to thank research teams from 12 sample sites for their hard work in the data collection. The authors also want to thank all the mothers who participated in this study.

For peer review only

Abstract

Objective: To explore and compare the predictors for exclusive breastfeeding (EBF) among migrant and non-migrant mothers in China.

Design: A large-scale cross-sectional study.

Setting: 12 counties/districts were covered in China.

Participants: A total number of 10,408 mothers were recruited, of which 3,571 mothers of 0-5 month-old-infants in urban China were used for analysis.

Outcome: The practice of EBF was calculated based on the foods and drinks consumed in the last 24-hours, as recommended by the World Health Organization.

Results: Around 30% of Chinese mothers with 0-5 month-old-infants, practiced EBF in urban areas, with no significant difference for migrant and non-migrant mothers (*P*=0.433). Among the migrant mothers, factors associated with EBF included residence in big cities [AOR 1.68 (1.20 - 2.34)], premature birth [AOR 0.27 (0.09 - 0.81)], knowledge on EBF [AOR 2.00 (1.51 - 2.65)], low intention of breastfeeding in the first month postpartum [AOR 0.59 (0.36 - 0.97)], and mothers working in agriculture-related fields or as casual workers [AOR 1.77 (1.18 – 2.64)]. Among non-migrant mothers, in addition to similar predictors including residence in big cities [AOR 1.40 (1.13 - 1.73)], knowledge on EBF [AOR 1.25 (1.02 - 1.53)] and low intention of breastfeeding in the first month postpartum [AOR 0.46 (0.31 - 0.70)], early initiation of breastfeeding (EIBF) [AOR 1.78 (1.35 - 2.33)] and cesarean delivery [AOR 0.74 (0.60 - 0.89)] were also factors associated with EBF.

Conclusions: There was no significant difference in the prevalence of EBF between migrant and non-migrant mothers in urban China. Premature birth and maternal occupation in agriculture-related fields or casual work were distinctive factors

For peer review only

associated with EBF for migrants, while EIBF and cesarean delivery were unique predictors for non-migrants.

Strengths and limitations of this study

- This is the first and largest study to explore and compare breastfeeding practices and its associate factors among migrant and non-migrant mothers in urban China.
- Mothers in large cities were more likely to practice EBF when compared with mothers in medium and small cities, regardless of their migrant status.
- The mothers covered in this study may not be representative of mothers in urban areas since we only invited mothers who brought their infants to immunization clinics to participate, which may overestimate the prevalence of exclusive breastfeeding.
- Since it is not a nationally representative study, the results cannot be generalized to all the urban areas of China.

For peer review only

Introduction

China has witnessed rapid economic growth in the past decades. Cross-country evidence showed that the prevalence of breastfeeding decreased with the increase in gross domestic product per capita[1]. Similar to the global trend, the prevalence of exclusive breastfeeding (EBF) in China dropped from 27.6% in 2008 to 20.7% in 2013[2,3]. Rapid economic development is inseparable from an increasing number of domestic migrants[4]. China has experienced an unprecedented internal migration in world history. On the one hand, better health infrastructures in cities can provide migrants with better health care services[5]. On the other hand, the household registration system and limited insurance coverage put migrants in a vulnerable situation with less potential access to urban benefits[6]. In addition to socio-economic and cultural differences between migrant and non-migrant women in China, previous studies also found that migrant women had poor knowledge and limited utilization of maternal health care services[7–9].

A large number of previous studies have focused on immigrant mothers, who moved to a country which was not their country of origin[10]. Immigrant mothers were found to be more likely to initiate and continue breastfeeding than non-immigrant mothers. However, EBF remained a challenge for both groups[10]. Little attention has been paid to breastfeeding practices and its associated factors for domestic migrant mothers. Previous literature in China disclosed the disparities in breastfeeding practices between urban and rural areas[3,11], employed and unemployed mothers[12], Han and other minority groups[13], and highly-educated and less-educated mothers[14]. A wide range of individual, socio-demographic, cultural, psychosocial and environmental factors had been identified as risk factors of exclusive breastfeeding in China[15,16], such as maternal and child characteristics[12,13,17–19], support from family members

For peer review only

For peer review only - http://bmjopen.bmj.com/site/about/guidelines.xhtml

and friends[18,20,21], maternity facility education, support and practice[11,17,19,22] as well as breastfeeding intention[18]. Yet, few studies have examined the difference in breastfeeding practice and its predictors among migrant and non-migrant mothers in China. To fill this knowledge gap, we analyzed large-scale cross-sectional survey data to examine the similarities and disparities in EBF and its associated factors, among migrant and non-migrant mothers in China.

Methods

Study design and participants

Data for this study was derived from a large-scale cross-sectional survey in China initiated by the China Development Research Foundation in 2017. This survey was designed by an independent team at the National Institute for Nutrition and Health of the Chinese Center for Disease Control and Prevention (NINH, China CDC). Calculation of sample size was based on the expected prevalence of EBF for 0-5 month-old-infants.

We adopted a multi-stage stratified cluster sampling approach while selecting the survey sample. In the first stage, all districts/counties were categorized into 4 strata, of which 12 districts/counties were selected (4 from large cities, 4 from medium and small cities, 2 from normal rural areas and 2 from poor rural areas). The selection of districts/counties considered their geographic distributions, executive capacities, collaboration of the provincial level CDC, and their population sizes. In the second stage, four clusters were randomly selected using the probability proportional to size sampling in each selected district/county. One cluster usually had one community health center. In the last stage, mothers who brought their 0-11 month-old-infants to immunization clinics, were randomly invited to participate in the survey until the

For peer review only

designed sample size was met. The inclusion criteria of this study were mothers of 0-11 month-old-infants who signed the informed consent form, had no psychiatric disorders, and were able to answer questions clearly. To decrease the reporting bias of feeding practice, we only invited mothers who were the primary caregivers of their infants. In total, 10,408 mothers of 0-11 month-old-infants were interviewed.

Patient and public involvement

This survey was undertaken by mothers of 0-11 month-old-infants in China to understand the prevalence of EBF and its associated factors. Neither these mothers nor the public were involved in the study design and implementation. The study results will be disseminated to the public through media briefings and scientific publications.

Data collection

This survey was conducted by the NINH, China CDC in collaboration with 12 teams at the provincial level CDC from September 2017 to January 2018. Data were collected through face-to-face interviews using a structured questionnaire programmed into smartphones or tablets. The questionnaire was developed by NINH, China CDC, which comprised eight sections: demographic characteristics, breastfeeding practices, maternal and child health, supportive environment for breastfeeding, health service, workplace, social environment and culture, and household financial situation related factors. The study design was approved by the Medical Ethics Committee at the NINH, China CDC. We obtained written consent forms from all mothers.

Statistical Analysis

For peer review only - http://bmjopen.bmj.com/site/about/guidelines.xhtml

We used the past 24 hours infant and young child feeding indicator method recommended by the World Health Organization (WHO) to assess the feeding practice of 0-5 month-old-infants to generate internationally comparable rates of exclusive breastfeeding. We defined the practice of being fed exclusively with breast milk in the last 24-hours as *exclusive breastfeeding*. Even though previous studies indicated that providing prelacteal feeds is a long-held tradition in many parts of China, regional disparities were quite large [23]. Additionally, water is the top one first drink received by the newborns in China and sometimes mothers revert to exclusive breastfeeding after breast milk came in [24]. Using the method can reduce the recall bias of mothers with older infants as well as the possibility of underestimating exclusive breastfeeding[24].

We first used descriptive analysis to report the selected characteristics, and then assessed their differences between migrant and non-migrant mothers, using Pearson's Chi-square test. Potential predictors were selected through reviewing relevant literature[3,12–15,18,25]. Chi-square tests were then used to examine the potential predictors of EBF for migrant and non-migrant mothers, respectively.

Socio-demographic characteristics included maternal age (<=25 years old, 26-35 years old and >=36 years old), maternal ethnicity (Han and others), maternal and paternal education (junior high school or less, high school, and college or higher education), maternal and paternal occupation (unemployed, agriculture-related or casual work, industry or business-related, professionals or white-collars), employment type of mothers (formal and informal) and place of residence (big cities, and small and medium cities). Obstetrical history comprised parity (1 and >=2), premature birth (yes and no), low birthweight (yes and no), mode of delivery (vaginal and cesarean) and delivery hospital type (hospitals at the municipal level or above level, hospitals at the

For peer review only - http://bmjopen.bmj.com/site/about/guidelines.xhtml

district/county level and others). Breastfeeding-related factors included early initiation of breastfeeding (EIBF) (yes and no), knowledge on EBF (yes and no), low intention of breastfeeding within 1 month postpartum (yes and no), and postpartum breastfeeding difficulties (yes and no). We defined EIBF as the practice of putting infants to the breast of their mothers within the first hour of birth based on the definition of the WHO [26]. We defined mothers who were always, very often or sometimes unwilling to breastfeed within 1 month postpartum as having a low intention of breastfeeding within 1 month postpartum. Social influence denoted whether her partner, friend, mother or mother-in-law agreed that breastmilk was better than breastmilk substitutes. Health-seeking behaviors comprised the timing of considering how to feed the infants and attending antenatal visits, breastfeeding education sessions or mother groups.

We then performed logistic regressions to identify predictors of EBF. The selection of covariate variables was based on the *p*-values of Chi-square tests ($P<0.05$). Odds ratios and 95% confidence intervals were displayed. The data analyses were performed by Stata version 15.0 (Stata Corporation, USA). We set the level of statistical significance at $\alpha=0.05$.

For peer review only

For peer review only - http://bmjopen.bmj.com/site/about/guidelines.xhtml

Results

Sample description and factors associated with EBF

Considering that migrants usually concentrate in urban areas, we only focused on 3,571 mothers of 0-5 month-old-infants who lived in big, medium, and small cities. Migrant mothers were defined as those who had resided in a specific county for a month or more and had been in a place different from their registered county. We excluded 172 observations due to the missing values of potential predictors, which left 3,399 (>95% of the original sample) observations for statistical analysis, of which 2,199 were non-migrants and 1,200 were migrants. A sensitivity analysis using the full sample revealed very similar migrant distribution to the one shown in this study.

Table 1 displayed the summary statistics and results of the bivariate analysis, reflecting significant differences in the distribution of selected characteristics and associated factors of EBF among non-migrant and migrant mothers. The prevalence of EBF was 30.3% among non-migrant mothers, which was not statistically different from 29.0% migrant mothers (P=0.433).

Migrant mothers were statistically different from non-migrant mothers in age, ethnicity, occupation (her and her partner's), formal employment status, and place of residence. More than 60% of mothers were aged 26-35 years in both groups, while the proportion of mothers above 36 years was significantly higher in non-migrant mothers (P<0.001). The likelihood of being of Han ethnicity, working as a professional or white-collar worker, and being formally employed was statistically higher in non-migrant mothers than migrant mothers as well (Table 1). Partners of non-migrant mothers were more likely to work as professionals or white-collar workers. For non-migrant mothers, socio-demographic characteristics like maternal education and occupation, paternal education, employment status of mothers, and place of residence were significantly

For peer review only - http://bmjopen.bmj.com/site/about/guidelines.xhtml

associated with EBF (P<0.001). For migrant mothers, in addition to maternal education and occupation, paternal education, employment status of mothers, and place of residence, the ethnicity of mothers also significantly affected the prevalence of EBF. Mothers or their partners with a college or higher education were more likely to practice EBF in both groups. The prevalence of EBF was higher in mothers who had a job, were formally employed, and lived in big cities among both migrant and non-migrant mothers. Mothers from the Han ethnic group were more likely to practice EBF than mothers from minority groups among the migrant group (Table 1).

Among factors about obstetrical history, the percentage of having only one child among migrant mothers was statistically higher than for non-migrant mothers (P<0.001). The proportions of premature birth and low birthweight, were quite similar between migrant and non-migrant mothers, while the rate of cesarean section was statistically higher in non-migrant mothers than migrant mothers. Cesarean section was significantly associated with a lower prevalence of EBF for both migrant and non-migrant mothers. However, for migrant women, premature birth and low birthweight of infants were also negatively related to EBF. A significantly larger proportion of migrant mothers delivered their infants in hospitals at the municipal level or above (P<0.001). Mothers who delivered infants in hospitals at the district/county level were less likely to practice breastfeeding in both groups.

EIBF, having a low intention of breastfeeding within 1 month postpartum and breastfeeding difficulties postpartum, occurred more frequently among migrant mothers than non-migrant mothers, while only the prevalence of having breastfeeding difficulties postpartum was statistically different between the two groups. Unlike the non-migrant group where EIBF was associated with EBF significantly (P<0.001), migrant mothers were more likely to practice EBF if they had breastfeeding difficulties

For peer review only - http://bmjopen.bmj.com/site/about/guidelines.xhtml

postpartum. More than half of the mothers knew the definition of EBF, and around 10% of them had a low intention of breastfeeding within 1 month postpartum in both groups. Mothers in both groups were more likely to practice EBF if they knew its definition and were less likely to do it if their intentions of breastfeeding were low within 1 month postpartum.

Even though more than 80% of mothers received positive influence from their partners, friends, mothers or mothers-in-law about breastmilk being better than breastmilk substitutes, these influences differed among migrant and non-migrant mothers. The influence from partner and friends was significantly associated with EBF for non-migrant mothers, while the influence from their partners and their own mothers were significantly associated with EBF for migrant mothers.

Non-migrant mothers were more likely to adopt health-seeking behaviors like considering how to feed their infants before pregnancy and attending antenatal visits, breastfeeding education sessions, and mother groups, than migrant mothers. Only the timing of considering how to feed their infants was statistically different between the two groups. Migrant mothers who considered how to feed their infants before pregnancy were more likely to practice EBF. Attending breastfeeding education sessions significantly affected the EBF practice for non-migrant mothers only.

For peer review only - http://bmjopen.bmj.com/site/about/guidelines.xhtml

Table 1 Factors associated with EBF practice among mothers of 0-5 month-old-infants by migration status

	Non-migrant mothers (n=2199)					Migrant mothers (n=1200)					
	Total		EBF			Total		EBF			
	N	%	n	%	*P*-value[b]	N	%	n	%	*P*-value[b]	*P*-value[a]
Socio-Demographic characteristics											
Maternal age					0.740					0.536	<0.001
<=25 years old	448	20.4	129	28.8		283	23.6	83	29.3		
26-35 years old	1411	64.2	432	30.6		799	66.6	236	29.5		
36+ years old	340	15.5	105	30.9		118	9.8	29	24.6		
Maternal ethnicity					0.080					0.019	0.002
Others	360	16.4	123	34.2		248	20.7	57	23.0		
Han	1839	83.6	543	29.5		952	79.3	291	30.6		
Maternal education					<0.001					<0.001	0.489
Junior high school or less	666	30.3	167	25.1		361	30.1	79	21.9		
High school	422	19.2	106	25.1		212	17.7	52	24.5		
College or higher education	1111	50.5	393	35.4		627	52.3	217	34.6		
Maternal occupation					<0.001					0.001	<0.001
Unemployed	776	35.3	201	25.9		449	37.4	101	22.5		
Agriculture-related or casual work	370	16.8	101	27.3		211	17.6	70	33.2		
Industry or business-related	325	14.8	106	32.6		237	19.8	82	34.6		
Professionals or white-collars	728	33.1	258	35.4		303	25.3	95	31.4		
Paternal education					<0.001					<0.001	0.211
Junior high school or less	696	31.7	174	25.0		345	28.8	80	23.2		
High school	404	18.4	90	22.3		233	19.4	54	23.2		
College or higher education	1099	50.0	402	36.6		622	51.8	214	34.4		
Paternal occupation					0.434					0.473	<0.001
Unemployed	112	5.1	37	33.0		49	4.1	11	22.4		
Agriculture-related or casual work	597	27.1	169	28.3		317	26.4	101	31.9		
Industry or business-related	660	30.0	195	29.5		453	37.8	127	28.0		
Professionals or white-collars	830	37.7	265	31.9		381	31.8	109	28.6		
Mother was formally employed					<0.001					<0.001	0.021
No	1356	61.7	365	26.9		788	65.7	197	25.0		
Yes	843	38.3	301	35.7		412	34.3	151	36.7		
Place of residence					<0.001					<0.001	<0.001

For peer review only - http://bmjopen.bmj.com/site/about/guidelines.xhtml

For peer review only

Small and medium sized cities	1222	55.6	299	24.5		411	34.3	82	20.0		
Big cities	977	44.4	367	37.6		789	65.8	266	33.7		
Obstetrical history											
Parity					0.875					0.105	<0.001
>=2	1181	53.7	356	30.1		509	42.4	135	26.5		
1	1018	46.3	310	30.5		691	57.6	213	30.8		
Premature birth					0.877					0.006	0.430
No	2112	96.0	639	30.3		1159	96.6	344	29.7		
Yes	87	4.0	27	31.0		41	3.4	4	9.8		
Low birthweight					0.389					0.019	0.807
No	2104	95.7	641	30.5		1146	95.5	340	29.7		
Yes	95	4.3	25	26.3		54	4.5	8	14.8		
Mode of delivery					<0.001					0.016	0.010
Vaginal	1329	60.4	438	33.0		779	64.9	244	31.3		
Cesarean	870	39.6	228	26.2		421	35.1	104	24.7		
Delivery hospital type					0.103					0.095	<0.001
Hospitals at the municipal or above level	1348	61.3	423	31.4		849	70.8	259	30.5		
Hospitals at the district/county level	796	36.2	222	27.9		316	26.3	77	24.4		
Others	55	2.5	21	38.2		35	2.9	12	34.3		
Breastfeeding related factors											
Early initiation of breastfeeding					<0.001					0.098	0.216
No	1917	87.2	538	28.1		1028	85.7	289	28.1		
Yes	282	12.8	128	45.4		172	14.3	59	34.3		
Knowledge on EBF					<0.001					<0.001	0.708
No	940	42.7	238	25.3		505	42.1	98	19.4		
Yes	1259	57.3	428	34.0		695	57.9	250	36.0		
Low intention of breastfeeding within 1 month postpartum					<0.001					0.029	0.115
No	2018	91.8	635	31.5		1082	90.2	324	29.9		
Yes	181	8.2	31	17.1		118	9.8	24	20.3		
Breastfeeding difficulties postpartum					0.436					<0.001	0.022
No	1567	71.3	467	29.8		810	67.5	210	25.9		
Yes	632	28.7	199	31.5		390	32.5	138	35.4		
Social influence											
Partner supports breastfeeding					0.012					0.031	0.868

For peer review only

No	270	12.3	64	23.7		145	12.1	31	21.4		
Yes	1929	87.7	602	31.2		1055	87.9	317	30.0		
Friend supports breastfeeding					0.031					0.217	0.719
No	318	14.5	80	25.2		179	14.9	45	25.1		
Yes	1881	85.5	586	31.2		1021	85.1	303	29.7		
Mother supports breastfeeding					0.295					0.014	0.259
No	255	11.6	70	27.5		155	12.9	32	20.6		
Yes	1944	88.4	596	30.7		1045	87.1	316	30.2		
Mother-in-law supports breastfeeding					0.740					0.198	0.114
No	312	14.2	92	29.5		147	12.3	36	24.5		
Yes	1887	85.8	574	30.4		1053	87.8	312	29.6		
Health-seeking behaviors											
Timing of considering how to feed the infants					0.490					0.002	<0.001
Before pregnancy	1208	54.9	365	30.2		532	44.3	178	33.5		
During pregnancy	646	29.4	188	29.1		429	35.8	119	27.7		
After birth	345	15.7	113	32.8		239	19.9	51	21.3		
Attend Antenatal visits					0.992					0.243	0.067
No	89	4.0	27	30.3		65	5.4	23	35.4		
Yes	2110	96.0	639	30.3		1135	94.6	325	28.6		
Attend breastfeeding education sessions					0.046					0.249	0.540
No	1101	50.1	312	28.3		614	51.2	169	27.5		
Yes	1098	49.9	354	32.2		586	48.8	179	30.5		
Attend mother groups					0.082					0.999	0.342
No	1373	62.4	434	31.6		769	64.1	223	29.0		
Yes	826	37.6	232	28.1		431	35.9	125	29.0		

[a] *P*-value of chi-square test to compare distribution of covariate variables between migrant and non-migrant mothers.
[b] *P*-value of chi-square test to identify risk factors of exclusive breastfeeding practice among migrant and non-migrant mothers respectively.

For peer review only

Predictors of breastfeeding practice among migrant and non-migrant mothers

We then performed logistic regression to determine the predictors of EBF, respectively. We only kept covariates with *p*-values smaller than 0.05 in Table 1.

Among socio-demographic factors, place of residence was significantly associated with EBF. Migrants living in big cities were more likely to practice EBF than those in small or medium cities (adjusted OR(AOR) =1.68, 95% CI 1.20 - 2.34) after adjusting for other potential confounders. The odds of EBF were lower among migrant mothers with preterm infants (AOR= 0.27, 95% CI 0.09 - 0.81) and having a low intention of breastfeeding in the first month postpartum (AOR= 0.59, 95% CI 0.36 - 0.97). Migrant mothers who worked in agriculture-related fields or as casual workers (AOR=1.77, 95% CI 1.18 - 2.64), and who knew EBF (AOR= 2.00, 95% CI 1.51 - 2.65) were more likely to practice EBF (Table 2).

Among non-migrant mothers, we found that residence in big cities (AOR=1.40, 95% CI 1.13 - 1.73), practicing EIBF (AOR=1.78, 95% CI 1.35 - 2.33), and knowledge on EBF (AOR=1.25, 95% CI 1.02 - 1.53), were significantly associated with optimal breastfeeding practice. However, cesarean section (AOR=0.74, 95% CI 0.60 - 0.89) and low intention of breastfeeding (AOR=0.46, 95% CI 0.31 - 0.70) significantly decreased the likelihood to practice EBF (Table 3).

For peer review only - http://bmjopen.bmj.com/site/about/guidelines.xhtml

Table 2 Predictors of EBF practice among migrant mothers of 0-5 month-old-infants (n=1200)

Risk factors	AOR	95% CI
Maternal ethnicity		
Others	1	
Han	1.14	(0.79 - 1.65)
Maternal education		
Junior high school or less	1	
High school	1.15	(0.72 - 1.83)
College or higher education	1.38	(0.83 - 2.28)
Maternal occupation		
Unemployed	1	
Agriculture-related or casual work	1.77**	(1.18 - 2.64)
Industry or business related	1.25	(0.84 - 1.86)
Professionals or white-collars	0.98	(0.61 - 1.55)
Paternal education		
Junior high school or less	1	
High school	0.71	(0.45 - 1.13)
College or higher education	0.90	(0.55 - 1.47)
Mather was formally employed		
No	1	
Yes	1.29	(0.88 - 1.91)
Place of residence		
Small and medium cities	1	
Big cities	1.68**	(1.20 - 2.34)
Premature birth		
No	1	
Yes	0.27*	(0.09 - 0.81)
Low birthweight		
No	1	
Yes	0.66	(0.29 - 1.52)
Mode of delivery		
Vaginal	1	
Cesarean	0.86	(0.65 - 1.14)
Knowledge on EBF		
No	1	
Yes	2.00***	(1.51 - 2.65)
Low intention of breastfeeding within 1 month postpartum		
No	1	
Yes	0.59*	(0.36 - 0.97)
Breastfeeding difficulties postpartum		
No	1	
Yes	1.32	(0.99 - 1.75)
Partner supports breastfeeding		
No	1	
Yes	1.34	(0.77 - 2.35)
Grandmother supports breastfeeding		
No	1	
Yes	1.51	(0.88 - 2.60)
Timing of considering how to feed the infants		
Before pregnancy	1	
During pregnancy	0.87	(0.65 - 1.17)
After birth	0.75	(0.51 - 1.11)

Odds ratios were adjusted for all the covariates in the model. * $P < 0.05$, ** $P < 0.01$, *** $P < 0.001$.

For peer review only

Table 3 Predictors of EBF practice among non-migrant mothers of 0-5 month-old-infants (n=2199)

Risk factors	AOR	95% CI
Maternal education		
Junior high school or less	1	
High school	0.98	(0.70 - 1.36)
College or higher education	0.98	(0.66 - 1.45)
Maternal occupation		
Unemployed	1	
Agriculture-related or casual work	1.15	(0.85 - 1.54)
Industry or business related	1.15	(0.84 - 1.59)
Professionals or white-collar	1.20	(0.85 - 1.70)
Paternal education		
Junior high school or less	1	
High school	0.81	(0.58 - 1.13)
College or higher education	1.33	(0.92 - 1.93)
Mather was formally employed		
No	1	
Yes	0.98	(0.72 - 1.35)
Place of residence		
Small and medium cities	1	
Big cities	1.40**	(1.13 - 1.73)
Mode of delivery		
Vaginal	1	
Cesarean	0.74**	(0.60 - 0.89)
Early initiation of breastfeeding		
No	1	
Yes	1.78***	(1.35 - 2.33)
Knowledge on EBF		
No	1	
Yes	1.25*	(1.02 - 1.53)
Low intention of breastfeeding within 1 month postpartum		
No	1	
Yes	0.46***	(0.31 - 0.70)
Partner supports breastfeeding		
No	1	
Yes	1.22	(0.84 - 1.78)
Friend supports breastfeeding		
No	1	
Yes	1.14	(0.81 - 1.61)
Attend breastfeeding education sessions		
No	1	
Yes	1.11	(0.92 - 1.36)

Odds ratios were adjusted for all the covariates in the model. * $P < 0.05$, ** $P < 0.01$, *** $P < 0.001$.

For peer review only

For peer review only - http://bmjopen.bmj.com/site/about/guidelines.xhtml

Discussion

In this study, we found that breastfeeding practice was suboptimal in both migrant and non-migrant mothers. The prevalence of EBF among non-migrant women was not significantly different from that of migrant mothers. This is the first and largest study to explore and compare breastfeeding practices and their potential predictors among migrant and non-migrant mothers in China. Multivariate regression results suggested that similar predictors of EBF for mothers in both groups included residence in big cities, knowledge on EBF, and low intention of breastfeeding in the first month postpartum. Mothers in big cities were approximately 1.5 times more likely to practice EBF. Among migrant mothers, having premature infants and working in agriculture-related fields or as casual workers, were associated with EBF practice. However, for non-migrant mothers, EIBF and cesarean delivery were unique predictors of EBF practice.

Living in big cities was the only common variable in demographic characteristics that were significantly associated with EBF practice in both groups. Previous literature also suggested a higher rate of EBF in big cities than in middle and small cities in China using nationally representative data derived from the Chinese National Nutrition and Health Survey conducted in 2013[3]. This is possibly due to better knowledge and supportive environment for mothers in big cities than those living in small and medium cities regardless of their migration status. For example, the proportion of mothers who received information on encouraging breastfeeding was higher in big cities (55.3%) than in small and medium cities (44.7%), and more than 92% of them received encouragement from the delivery hospital in big cities. This revealed that both migrant and non-migrant mothers may benefit from better breastfeeding-related education and support, in big cities in China.

Another common predictor of EBF for both migrant and non-migrant mothers was knowledge on EBF. Some of the previous studies used knowledge scores on breastfeeding benefits as a predictor of breastfeeding practice, but they failed to provide consistent evidence

For peer review only - http://bmjopen.bmj.com/site/about/guidelines.xhtml

on its association with EBF[11,18]. However, we only focused on the key message that infants should be exclusively breastfed for the first 6 months. This implies that future education intervention projects should pay more attention to disseminating core messages in promoting optimal breastfeeding practices.

Additionally, we also found that having a low intention of breastfeeding in the first month postpartum significantly decreased the odds of practicing EBF for both groups. Cracked nipples or nipple pain, and insufficient breastmilk supply ranked as the top two reasons, which together accounted for around half of all the answers causing low breastfeeding intention. In alignment with previous studies, nipple pain or fissure within the first month postpartum was the commonest reason for the low intention of breastfeeding among mothers regardless of their migration status[27,28]. Given that nipple pain was often attributed to incorrect positioning and attachment[28], health care providers need to find effective measures to help establish proper positioning for mothers, good attachment of the babies to the breast as well as effective sucking. In addition, insufficient milk supply was frequently reported by previous studies as the leading cause of breastfeeding cessation or its exclusivity[29,30]. However, there is limited evidence on how to design interventions to address maternal perceptions or concerns of insufficient milk supply[31,32]. Further studies are needed to identify effective interventions regarding the maternal perception of milk supply.

In addition to the common predictors of EBF, as explained above, there were unique risk factors for migrant and non-migrant mothers, respectively. Among migrant mothers, having premature infants significantly decreased the prevalence of EBF. The EBF rate for migrant mothers with preterm infants was 9.8%, which was vastly lower than that of non-migrant mothers (31.0%). The low EBF rate could be attributable to mother-infant separation in neonatal intensive care units (NICUs) in China[33], which resulted in many difficulties and challenges for mothers with premature infants. The disparity in the EBF rate of premature

For peer review only - http://bmjopen.bmj.com/site/about/guidelines.xhtml

infants may come from the different choices of hospitalization services between migrant and non-migrant mothers. Even though migrant mothers are more likely to deliver their infants in higher level hospitals in general, the proportion of migrant mothers with premature infants who delivered in hospitals at the municipal or higher levels was 65.9%, lower than that of non-migrant mothers (73.6%). In China, high-level hospitals are believed to have well-trained health workers and high-quality medical care [34]. An internationally well-known practice such as Kangaroo Care (KC), has only been implemented in some NICUs of high-level hospitals in China as pilot studies, for promoting the development of premature infants [35]. Implementation of KC was hindered by the limits on parental visitation, inconsistent guidelines, and safety fears of the medical staff [35]. Medical practice in NICUs may need to encourage more involvement of parents to care for their infants.

Additionally, migrant mothers working in agriculture-related fields or as casual workers were more likely to practice EBF. This is possibly because when compared with mothers working in other fields, they have more flexible time, and the shortest commuting time of 29-minutes for those who have already gone back to work. When compared with unemployed mothers, these migrant mothers were more likely to adopt health-seeking behaviors like starting to consider how to feed their infants before pregnancy and attending breastfeeding education sessions in hospitals. These findings were in alignment with previous studies, finding that mothers who worked full-time were less likely to initiate or continue breastfeeding[36]. Our results suggested that working was not necessarily a barrier to breastfeeding. Allowing lactating mothers to work with a flexible schedule that may provide support for breastfeeding.

Among non-migrant mothers, EIBF and cesarean section were distinctive predictors of EBF. The rate of EIBF was 12.8% in non-migrant mothers, which was a little higher than the 9% found in a study in the Sichuan province[37], but much lower than the national

For peer review only - http://bmjopen.bmj.com/site/about/guidelines.xhtml

prevalence of 28.7% in 2013[38] and the 59.4% found in a study in central and western China[39]. A large regional disparity of EIBF in China may be due to the delayed process of implementing Early Essential Newborn Care (EENC) in China. Even in places where EENC had been implemented, skin-to-skin contact was often interrupted due to inadequate facilities and early initiation of breastfeeding was difficult to be ensured[40]. Hospitals in China need to speed up their process of adopting EENC recommended practices and national policies are needed to be established to help hospitals and health workers overcome obstacles in implementing these practices.

For obstetrical history-related factors, we also found that the prevalence of cesarean delivery was high and negatively associated with EBF among non-migrant mothers only. The prevalence of cesarean delivery was 39.6% among non-migrant mothers, which was higher than the national average of 36.7% in 2018[41]. Even though China had made efforts in decreasing cesarean delivery in the past decade[42], the national prevalence is still far higher than the suggested prevalence of 10-15% by the WHO[43]. The government and health facilities need to make effective policies and measures in addressing non-clinical reasons for preferring a cesarean delivery, such as maternal request and perceived convenience[44].

This study derived data from a large-scale cross-sectional survey covering 8 sample sites in urban areas, which is the first and largest study to explore and compare breastfeeding practices and their potential predictors among migrant and non-migrant mothers in China. Findings in this study filled the knowledge gap in the similarities and differences in the predictors of EBF among migrant and non-migrant mothers, which had important implications for future studies as well as health interventions to promote optimal breastfeeding practices in China.

However, our study still faced the following limitations. First, differences in the predictors of EBF among migrant and non-migrant mothers revealed in this study, may come

For peer review only

from different choices in hospitalization services. Migrant mothers may have restricted access to better health services due to social exclusions, lack of social networks, and health insurance. Future studies need to take a closer look at the differences in health facility choices among migrant and non-migrant mothers and identify potential obstacles faced by the migrants. Second, mothers covered in this study may not be representative of the mothers in general. That is because we only interviewed mothers who brought their infants into immunization clinics at the time of the interview, which naturally excluded children who were left behind in their early childhood and may overestimate the prevalence of EBF for both groups [45,46]. A low rate of EBF would be really problematic since it will not only result in nutrition-related harm to the early childhood development of children, but also increase the health risks of mothers in China[1]. Third, when selecting sample sites in the first stage, we considered the executive capacities, and collaboration of the provincial level CDC. Thus, sample sites selected in this study to some extent reflected a higher efficiency of health systems at the county/district level. Caution is needed when interpreting these results and the results disclosed in this study cannot be generalized to the whole urban areas of China.

To conclude, this study found that there was no significant difference in the prevalence of EBF between migrant and non-migrant mothers in our sampled urban areas of China. Even though the predictors of EBF shared some similarities between migrant and non-migrant mothers, future interventions may still need to adopt different strategies in promoting optimal breastfeeding practices in different groups.

For peer review only - http://bmjopen.bmj.com/site/about/guidelines.xhtml

Reference

1 Victora CG, Bahl R, Barros AJD, *et al.* Breastfeeding in the 21st century: epidemiology, mechanisms, and lifelong effect. *Lancet* 2016;**387**:475–90. doi:10.1016/S0140-6736(15)01024-7

2 Center for Health Statistics and Information at Ministry of Health of People's Republic of China. *An Analysis Report of National Health Services Survey in China,2008*. Beijing: 2009.

3 Duan Y, Yang Z, Lai J, *et al.* Exclusive Breastfeeding Rate and Complementary Feeding Indicators in China: A National Representative Survey in 2013. *Nutrients* 2018;**10**:249. doi:10.3390/nu10020249

4 Wang X-R, Hui EC-M, Sun J-X. Population migration, urbanization and housing prices: Evidence from the cities in China. *Habitat Int* 2017;**66**:49–56. doi:10.1016/j.habitatint.2017.05.010

5 Song Y, Sun W. Health Consequences of Rural-to-Urban Migration: Evidence from Panel Data in China. *Health Econ* 2016;**25**:1252–67. doi:10.1002/hec.3212

6 Peng Y, Chang W, Zhou H, *et al.* Factors associated with health-seeking behavior among migrant workers in Beijing, China. *BMC Health Serv Res* 2010;**10**:69. doi:10.1186/1472-6963-10-69

7 Zhao Q, Kulane A, Gao Y, *et al.* Knowledge and attitude on maternal health care among rural-to-urban migrant women in Shanghai, China. *BMC Womens Health* Published Online First: 2009. doi:10.1186/1472-6874-9-5

8 Zhao Q, Huang ZJ, Yang S, *et al.* The utilization of antenatal care among rural-to-urban migrant women in Shanghai: A hospital-based cross-sectional study. *BMC Public Health* Published Online First: 2012. doi:10.1186/1471-2458-12-

For peer review only

1012

9 WEI X, PEARSON S, ZHANG Z, *et al.* COMPARING KNOWLEDGE AND USE OF HEALTH SERVICES OF MIGRANTS FROM RURAL AND URBAN AREAS IN KUNMING CITY, CHINA. *J Biosoc Sci* 2010;**42**:743–56. doi:10.1017/S0021932010000313

10 Dennis C, Shiri R, Brown HK, *et al.* Breastfeeding rates in immigrant and non-immigrant women: A systematic review and meta-analysis. *Matern Child Nutr* 2019;**15**:e12809. doi:10.1111/mcn.12809

11 Gao H, Wang Q, Hormann E, *et al.* Breastfeeding practices on postnatal wards in urban and rural areas of the Deyang region, Sichuan province of China. *Int Breastfeed J* 2016;**11**:11. doi:10.1186/s13006-016-0070-0

12 Li L, Li S, Ali M, *et al.* Feeding practice of infants and their correlates in urban areas of Beijing, China. *Pediatr Int* 2003;**45**:400–6. doi:10.1046/j.1442-200X.2003.01754.x

13 Xu F, Binns C, Nazi G, *et al.* A comparison of breastfeeding among Han, Uygur and other ethnic groups in Xinjiang, PR China. *BMC Public Health* 2006;**6**:196. doi:10.1186/1471-2458-6-196

14 Zhao J, Zhao Y, Du M, *et al.* Maternal education and breastfeeding practices in China: A systematic review and meta-analysis. *Midwifery* 2017;**50**:62–71. doi:10.1016/j.midw.2017.03.011

15 Rollins NC, Bhandari N, Hajeebhoy N, *et al.* Why invest, and what it will take to improve breastfeeding practices? *Lancet* 2016;**387**:491–504. doi:10.1016/S0140-6736(15)01044-2

16 Li J, Nguyen TT, Wang X, *et al.* Breastfeeding practices and associated factors at the individual, family, health facility and environmental levels in China.

For peer review only - http://bmjopen.bmj.com/site/about/guidelines.xhtml

Manteral Child Nutr 2020.

17 Waits A, Guo CY, Chien LY. Evaluation of factors contributing to the decline in exclusive breastfeeding at 6 months postpartum: The 2011-2016 National Surveys in Taiwan. *Birth* Published Online First: 2018. doi:10.1111/birt.12340

18 Ling Shi, Jingxu Zhang, Yan Wang, *et al.* Breastfeeding in Rural China: Association Between Knowledge, Attitudes, and Practices. *J Hum Lact* 2008;**24**:377–85. doi:10.1177/0890334408319157

19 Qiu L, Zhao Y, Binns CW, *et al.* Initiation of breastfeeding and prevalence of exclusive breastfeeding at hospital discharge in urban, suburban and rural areas of Zhejiang China. *Int Breastfeed J* 2009;**4**:1. doi:10.1186/1746-4358-4-1

20 Su M, Ouyang YQ. Father's role in breastfeeding promotion: Lessons from a quasi-experimental trial in China. *Breastfeed Med* Published Online First: 2016. doi:10.1089/bfm.2015.0144

21 Ke J, Ouyang Y-Q, Redding SR. Family-Centered Breastfeeding Education to Promote Primiparas' Exclusive Breastfeeding in China. *J Hum Lact* 2018;**34**:365–78. doi:10.1177/0890334417737293

22 Huang P, Ren J, Liu Y, *et al.* Factors affecting breastfeeding adherence among Chinese mothers: A multicenter study. *Med (United States)* Published Online First: 2017. doi:10.1097/MD.0000000000007619

23 Tang L, Hewitt K, Yu C. Prelacteal Feeds in China. *Curr Pediatr Rev* 2012;**8**:304–12. doi:10.2174/157339612803307688

24 Greiner T. Exclusive breastfeeding: measurement and indicators. *Int Breastfeed J* 2014;**9**:18. doi:10.1186/1746-4358-9-18

25 Dennis C-L, Gagnon A, Van Hulst A, *et al.* Prediction of Duration of Breastfeeding among Migrant and Canadian-Born Women: Results from a

For peer review only - http://bmjopen.bmj.com/site/about/guidelines.xhtml

Multi-Center Study. *J Pediatr* 2013;**162**:72–9. doi:10.1016/j.jpeds.2012.06.041

26 Daelmans B, Dewey K, Arimond M. New and Updated Indicators for Assessing Infant and Young Child Feeding. *Food Nutr Bull* 2009;**30**:S256–62. doi:10.1177/15648265090302S210

27 McCann MF, Baydar N, Williams RL. Breastfeeding Attitudes and Reported Problems in a National Sample of WIC Participants. *J Hum Lact* 2007;**23**:314–24. doi:10.1177/0890334407307882

28 Kent J, Ashton E, Hardwick C, *et al.* Nipple Pain in Breastfeeding Mothers: Incidence, Causes and Treatments. *Int J Environ Res Public Health* 2015;**12**:12247–63. doi:10.3390/ijerph121012247

29 Balogun OO, Dagvadorj A, Anigo KM, *et al.* Factors influencing breastfeeding exclusivity during the first 6 months of life in developing countries: a quantitative and qualitative systematic review. *Matern Child Nutr* 2015;**11**:433–51. doi:10.1111/mcn.12180

30 Flaherman VJ, Beiler JS, Cabana MD, *et al.* Relationship of newborn weight loss to milk supply concern and anxiety: the impact on breastfeeding duration. *Matern Child Nutr* 2016;**12**:463–72. doi:10.1111/mcn.12171

31 Galipeau R, Baillot A, Trottier A, *et al.* Effectiveness of interventions on breastfeeding self-efficacy and perceived insufficient milk supply: A systematic review and meta-analysis. *Matern Child Nutr* 2018;**14**. doi:10.1111/mcn.12607

32 Wood NK, Sanders EA, Lewis FM, *et al.* Pilot test of a home-based program to prevent perceived insufficient milk. *Women and Birth* 2017;**30**:472–80. doi:10.1016/j.wombi.2017.04.006

33 Wang Y, Briere C-E, Xu W, *et al.* Factors Affecting Breastfeeding Outcomes

For peer review only

For peer review only - http://bmjopen.bmj.com/site/about/guidelines.xhtml

at Six Months in Preterm Infants. *J Hum Lact* 2019;**35**:80–9. doi:10.1177/0890334418771307

34 Wang H, Zhang D, Hou Z, *et al.* Association between social health insurance and choice of hospitals among internal migrants in China: a national cross-sectional study. *BMJ Open* 2018;**8**:e018440. doi:10.1136/bmjopen-2017-018440

35 Zhang Y, Deng Q, Zhu B, *et al.* Neonatal intensive care nurses' knowledge and beliefs regarding kangaroo care in China: a national survey. *BMJ Open* 2018;**8**:e021740. doi:10.1136/bmjopen-2018-021740

36 Mandal B, Roe BE, Fein SB. The differential effects of full-time and part-time work status on breastfeeding. *Health Policy (New York)* 2010;**97**:79–86. doi:10.1016/j.healthpol.2010.03.006

37 Tang L, Binns CW, Lee AH, *et al.* Low Prevalence of Breastfeeding Initiation within the First Hour of Life in a Rural Area of Sichuan Province, China. *Birth* 2013;**40**:134–42. doi:10.1111/birt.12038

38 UNICEF. Children in China: An Atlas of Social Indicators of Children in China 2018. Beijing: 2018.

39 Guo S, Fu X, Scherpbier RW, *et al.* Breastfeeding rates in central and western China in 2010: implications for child and population health. *Bull World Health Organ* 2013;**91**:322–31. doi:10.2471/BLT.12.111310

40 Qu W, Yue Q, Wang Y, *et al.* Implementation of the Early Essential Newborn Care (EENC) on neonatal outcomes in west China: an observational study. *Lancet* 2018;**392**:S54. doi:10.1016/S0140-6736(18)32683-7

41 Li H, Hellerstein S, Zhou Y, *et al.* Trends in Cesarean Delivery Rates in China, 2008-2018. *JAMA* 2020;**323**:89. doi:10.1001/jama.2019.17595

For peer review only

42 Liang J, Mu Y, Li X, *et al.* Relaxation of the one child policy and trends in caesarean section rates and birth outcomes in China between 2012 and 2016: observational study of nearly seven million health facility births. *BMJ* 2018;:k817. doi:10.1136/bmj.k817

43 Betran A, Torloni M, Zhang J, *et al.* WHO Statement on Caesarean Section Rates. *BJOG An Int J Obstet Gynaecol* 2016;**123**:667–70. doi:10.1111/1471-0528.13526

44 Long Q, Kingdon C, Yang F, *et al.* Prevalence of and reasons for women's, family members', and health professionals' preferences for cesarean section in China: A mixed-methods systematic review. *PLOS Med* 2018;**15**:e1002672. doi:10.1371/journal.pmed.1002672

45 Tian X, Ding C, Shen C, *et al.* Does Parental Migration Have Negative Impact on the Growth of Left-Behind Children?—New Evidence from Longitudinal Data in Rural China. *Int J Environ Res Public Health* 2017;**14**:1308. doi:10.3390/ijerph14111308

46 Zhang N, Bécares L, Chandola T. Does the timing of parental migration matter for child growth? A life course study on left-behind children in rural China. *BMC Public Health* 2015;**15**:966. doi:10.1186/s12889-015-2296-y

For peer review only

STROBE Statement—Checklist of items that should be included in reports of ***cross-sectional studies***

	Item No	Page number in manuscript	Recommendation
**Title and abstract**	1	Page 3	(*a*) Indicate the study's design with a commonly used term in the title or the abstract
		Page 3	(*b*) Provide in the abstract an informative and balanced summary of what was done and what was found
**Introduction**			
Background/rationale	2	Page 5	Explain the scientific background and rationale for the investigation being reported
Objectives	3	Page 5	State specific objectives, including any prespecified hypotheses
**Methods**			
Study design	4	Page 6	Present key elements of study design early in the paper
Setting	5	Page 6-7	Describe the setting, locations, and relevant dates, including periods of recruitment, exposure, follow-up, and data collection
Participants	6	Page 6-7	(*a*) Give the eligibility criteria, and the sources and methods of selection of participants
Variables	7	Page 8	Clearly define all outcomes, exposures, predictors, potential confounders, and effect modifiers. Give diagnostic criteria, if applicable
Data sources/ measurement	8*	Page 8	For each variable of interest, give sources of data and details of methods of assessment (measurement). Describe comparability of assessment methods if there is more than one group
Bias	9	Page 8	Describe any efforts to address potential sources of bias
Study size	10	Page 6	Explain how the study size was arrived at
Quantitative variables	11	Page 8	Explain how quantitative variables were handled in the analyses. If applicable, describe which groupings were chosen and why
Statistical methods	12	Page 8-9	(*a*) Describe all statistical methods, including those used to control for confounding
		Page 9	(*b*) Describe any methods used to examine subgroups and interactions
		Page 10	(*c*) Explain how missing data were addressed
		Not applicable	(*d*) If applicable, describe analytical methods taking account of sampling strategy
		Page 10	(*e*) Describe any sensitivity analyses
**Results**			
Participants	13*	Page 10	(a) Report numbers of individuals at each stage of study—eg numbers potentially eligible, examined for eligibility, confirmed eligible, included in the study, completing follow-up, and analysed
		Not applicable	(b) Give reasons for non-participation at each stage
		Not applicable	(c) Consider use of a flow diagram
Descriptive data	14*	Page 10-12	(a) Give characteristics of study participants (eg demographic, clinical, social) and information on exposures and potential confounders
		Not applicable	(b) Indicate number of participants with missing data for each variable of interest

For peer review only - http://bmjopen.bmj.com/site/about/guidelines.xhtml

Outcome data	15*	Page 10	Report numbers of outcome events or summary measures
Main results	16	Page 10-18	(*a*) Give unadjusted estimates and, if applicable, confounder-adjusted estimates and their precision (eg, 95% confidence interval). Make clear which confounders were adjusted for and why they were included
		Not applicable	(*b*) Report category boundaries when continuous variables were categorized
		Not applicable	(*c*) If relevant, consider translating estimates of relative risk into absolute risk for a meaningful time period
Other analyses	17	Page 16-18	Report other analyses done—eg analyses of subgroups and interactions, and sensitivity analyses
**Discussion**			
Key results	18	Page 19	Summarise key results with reference to study objectives
Limitations	19	Page 22-23	Discuss limitations of the study, taking into account sources of potential bias or imprecision. Discuss both direction and magnitude of any potential bias
Interpretation	20	Page 19-22	Give a cautious overall interpretation of results considering objectives, limitations, multiplicity of analyses, results from similar studies, and other relevant evidence
Generalisability	21	Page 22-23	Discuss the generalisability (external validity) of the study results
**Other information**			
Funding	22	Title page	Give the source of funding and the role of the funders for the present study and, if applicable, for the original study on which the present article is based

*Give information separately for exposed and unexposed groups.

Note: An Explanation and Elaboration article discusses each checklist item and gives methodological background and published examples of transparent reporting. The STROBE checklist is best used in conjunction with this article (freely available on the Web sites of PLoS Medicine at http://www.plosmedicine.org/, Annals of Internal Medicine at http://www.annals.org/, and Epidemiology at http://www.epidem.com/). Information on the STROBE Initiative is available at www.strobe-statement.org.

For peer review only

BMJ Open

Predictors of exclusive breastfeeding practice among migrant and non-migrant mothers in urban China: results from a cross-sectional survey

Journal:	*BMJ Open*
Manuscript ID	bmjopen-2020-038268.R3
Article Type:	Original research
Date Submitted by the Author:	16-Jul-2020
Complete List of Authors:	Li, Jia; Tsinghua University School of Social Sciences, ; China Development Research Foundation, Duan, Yifan; Chinese Center for Disease Control and Prevention National Institute for Nutrition and Health Bi, Ye; Chinese Center for Disease Control and Prevention National Institute for Nutrition and Health Wang, Jie; Chinese Center for Disease Control and Prevention National Institute for Nutrition and Health Lai, Jianqiang; Chinese Center for Disease Control and Prevention National Institute for Nutrition and Health Zhao, Chen; China Development Research Foundation Fang, Jin; China Development Research Foundation Yang, Zhenyu; Chinese Center for Disease Control and Prevention National Institute for Nutrition and Health
**Primary Subject Heading**:	Public health
Secondary Subject Heading:	Epidemiology
Keywords:	NUTRITION & DIETETICS, PUBLIC HEALTH, EPIDEMIOLOGY

SCHOLARONE™
Manuscripts

I, the Submitting Author has the right to grant and does grant on behalf of all authors of the Work (as defined in the below author licence), an exclusive licence and/or a non-exclusive licence for contributions from authors who are: i) UK Crown employees; ii) where BMJ has agreed a CC-BY licence shall apply, and/or iii) in accordance with the terms applicable for US Federal Government officers or employees acting as part of their official duties; on a worldwide, perpetual, irrevocable, royalty-free basis to BMJ Publishing Group Ltd ("BMJ") its licensees and where the relevant Journal is co-owned by BMJ to the co-owners of the Journal, to publish the Work in this journal and any other BMJ products and to exploit all rights, as set out in our licence.

The Submitting Author accepts and understands that any supply made under these terms is made by BMJ to the Submitting Author unless you are acting as an employee on behalf of your employer or a postgraduate student of an affiliated institution which is paying any applicable article publishing charge ("APC") for Open Access articles. Where the Submitting Author wishes to make the Work available on an Open Access basis (and intends to pay the relevant APC), the terms of reuse of such Open Access shall be governed by a Creative Commons licence – details of these licences and which Creative Commons licence will apply to this Work are set out in our licence referred to above.

Other than as permitted in any relevant BMJ Author's Self Archiving Policies, I confirm this Work has not been accepted for publication elsewhere, is not being considered for publication elsewhere and does not duplicate material already published. I confirm all authors consent to publication of this Work and authorise the granting of this licence.

For peer review only

Predictors of exclusive breastfeeding practice among migrant and non-migrant mothers in urban China: results from a cross-sectional survey

Running title: Exclusive breastfeeding in urban China

Jia Li, PhD[1,2], Yifan Duan, MSc[3], Ye Bi, MSc[3], Jie Wang, PhD[3], Jianqiang Lai, PhD[3], Chen Zhao, MSocsc[2], Jin Fang, PhD[2], Zhenyu Yang, PhD[3,*]

[1] Institute of Economics, School of Social Sciences, Tsinghua University, Beijing, China

[2] China Development Research Foundation, Beijing, China

[3] National Institute for Nutrition and Health, Chinese Center for Disease Control and Prevention, Beijing, China

[*] Correspondence and reprint request to: Dr Zhenyu Yang (yangzy@ninh.chinacdc.cn), National Institute for Nutrition and Health, Chinese Center for Disease Control and Prevention, No.27 Nanwei Road, Xicheng District, Beijing, China, 100050, Phone +86-10-66237198, Fax +86-10-67791992

**Manuscript statistics:**	Number of tables: 3
Main text word count: 3796	Number of figures: 0
Abstract word count: 277	Number of references: 46

Disclaimers

The views expressed in the article are those of the authors and do not necessarily reflect the views of the institution and funder.

For peer review only

Funding source

The study was funded by the Bill & Melinda Gates Foundation to China Development Research Foundation (Grant Number OPP1152715).

Competing interests

None of the authors had a conflict of interest related to any part of this study or manuscript.

Authors' contributions

J.L., Y.D., Z.Y. and J.L designed the study; Y.D., Y.B., J.W. and Z.Y. supervised the field study; J.L., Y.D. and Z.Y. acquired and analyzed the data; J.L. interpreted the data and drafted the first manuscript; and Z.Y., Y.D., C.Z. and F.J. provided critical intellectual feedback to help revise the manuscript. All authors have read and approved the final manuscript.

Availability of data and materials

This dataset is available from the corresponding author on reasonable request.

Patient consent for publication

Not required

Disclosure of prior presentation of study data as an abstract or poster

None

Acknowledgements

The authors would like to thank research teams from 12 sample sites for their hard work in the data collection. The authors also want to thank all the mothers who participated in this study.

For peer review only - http://bmjopen.bmj.com/site/about/guidelines.xhtml

Abstract

Objective: To explore and compare the predictors for exclusive breastfeeding (EBF) among migrant and non-migrant mothers in China.

Design: A large-scale cross-sectional study.

Setting: 12 counties/districts were covered in China.

Participants: A total number of 10,408 mothers were recruited, of which 3,571 mothers of 0-5 month-old-infants in urban China were used for analysis.

Outcome: The practice of EBF was calculated based on the foods and drinks consumed in the last 24-hours, as recommended by the World Health Organization.

Results: Around 30% of Chinese mothers with 0-5 month-old-infants, practiced EBF in urban areas, with no significant difference for migrant and non-migrant mothers (P=0.433). Among the migrant mothers, factors associated with EBF included residence in big cities [AOR 1.68 (1.20 - 2.34)], premature birth [AOR 0.27 (0.09 - 0.81)], knowledge on EBF [AOR 2.00 (1.51 - 2.65)], low intention of breastfeeding in the first month postpartum [AOR 0.59 (0.36 - 0.97)], and mothers working in agriculture-related fields or as casual workers [AOR 1.77 (1.18 – 2.64)]. Among non-migrant mothers, in addition to similar predictors including residence in big cities [AOR 1.40 (1.13 - 1.73)], knowledge on EBF [AOR 1.25 (1.02 - 1.53)] and low intention of breastfeeding in the first month postpartum [AOR 0.46 (0.31 - 0.70)], early initiation of breastfeeding (EIBF) [AOR 1.78 (1.35 - 2.33)] and cesarean delivery [AOR 0.74 (0.60 - 0.89)] were also factors associated with EBF.

Conclusions: There was no significant difference in the prevalence of EBF between migrant and non-migrant mothers in urban China. Premature birth and maternal occupation in agriculture-related fields or casual work were distinctive factors

For peer review only - http://bmjopen.bmj.com/site/about/guidelines.xhtml

associated with EBF for migrants, while EIBF and cesarean delivery were unique predictors for non-migrants.

Strengths and limitations of this study

- This is the first and largest study to explore and compare breastfeeding practices and its associate factors among migrant and non-migrant mothers in urban China.
- Mothers in large cities were more likely to practice EBF when compared with mothers in medium and small cities, regardless of their migrant status.
- The mothers covered in this study may not be representative of mothers in urban areas since we only invited mothers who brought their infants to immunization clinics to participate, which may overestimate the prevalence of exclusive breastfeeding.
- Since it is not a nationally representative study, the results cannot be generalized to all the urban areas of China.

For peer review only

For peer review only - http://bmjopen.bmj.com/site/about/guidelines.xhtml

Introduction

China has witnessed rapid economic growth in the past decades. Cross-country evidence showed that the prevalence of breastfeeding decreased with the increase in gross domestic product per capita[1]. Similar to the global trend, the prevalence of exclusive breastfeeding (EBF) in China dropped from 27.6% in 2008 to 20.7% in 2013[2,3]. Rapid economic development is inseparable from an increasing number of domestic migrants[4]. China has experienced an unprecedented internal migration in world history. On the one hand, better health infrastructures in cities can provide migrants with better health care services[5]. On the other hand, the household registration system and limited insurance coverage put migrants in a vulnerable situation with less potential access to urban benefits[6]. In addition to socio-economic and cultural differences between migrant and non-migrant women in China, previous studies also found that migrant women had poor knowledge and limited utilization of maternal health care services[7–9].

A large number of previous studies have focused on immigrant mothers, who moved to a country that was not their country of origin[10]. Immigrant mothers were found to be more likely to initiate and continue breastfeeding than non-immigrant mothers. However, EBF remained a challenge for both groups[10]. Little attention has been paid to breastfeeding practices and its associated factors for domestic migrant mothers. Previous literature in China disclosed the disparities in breastfeeding practices between urban and rural areas[3,11], employed and unemployed mothers[12], Han and other minority groups[13], and highly-educated and less-educated mothers[14]. A wide range of individual, socio-demographic, cultural, psychosocial and environmental factors had been identified as risk factors of exclusive breastfeeding in China[15,16], such as maternal and child characteristics[12,13,17–19], support from family members

For peer review only - http://bmjopen.bmj.com/site/about/guidelines.xhtml

For peer review only

and friends[18,20,21], maternity facility education, support and practice[11,17,19,22] as well as breastfeeding intention[18]. Yet, few studies have examined the difference in breastfeeding practice and its predictors among migrant and non-migrant mothers in China. To fill this knowledge gap, we analyzed large-scale cross-sectional survey data to examine the similarities and disparities in EBF and its associated factors, among migrant and non-migrant mothers in China.

Methods

Study design and participants

Data for this study was derived from a large-scale cross-sectional survey in China initiated by the China Development Research Foundation in 2017. This survey was designed by an independent team at the National Institute for Nutrition and Health of the Chinese Center for Disease Control and Prevention (NINH, China CDC). Calculation of sample size was based on the expected prevalence of EBF for 0-5 month-old-infants.

We adopted a multi-stage stratified cluster sampling approach while selecting the survey sample. In the first stage, all districts/counties were categorized into 4 strata, of which 12 districts/counties were selected (4 from large cities, 4 from medium and small cities, 2 from normal rural areas and 2 from poor rural areas). The selection of districts/counties considered their geographic distributions, executive capacities, collaboration of the provincial level CDC, and their population sizes. In the second stage, four clusters were randomly selected using the probability proportional to size sampling in each selected district/county. One cluster usually had one community health center. In the last stage, mothers who brought their 0-11 month-old-infants to immunization clinics, were randomly invited to participate in the survey until the

For peer review only

designed sample size was met. The inclusion criteria of this study were mothers of 0-11 month-old-infants who signed the informed consent form, had no psychiatric disorders, and were able to answer questions clearly. To decrease the reporting bias of feeding practice, we only invited mothers who were the primary caregivers of their infants. In total, 10,408 mothers of 0-11 month-old-infants were interviewed.

Patient and public involvement

This survey was undertaken by mothers of 0-11 month-old-infants in China to understand the prevalence of EBF and its associated factors. Neither these mothers nor the public were involved in the study design and implementation. The study results will be disseminated to the public through media briefings and scientific publications.

Data collection

This survey was conducted by the NINH, China CDC in collaboration with 12 teams at the provincial level CDC from September 2017 to January 2018. Data were collected through face-to-face interviews using a structured questionnaire programmed into smartphones or tablets. The questionnaire was developed by NINH, China CDC, which comprised eight sections: demographic characteristics, breastfeeding practices, maternal and child health, supportive environment for breastfeeding, health service, workplace, social environment and culture, and household financial situation related factors. The study design was approved by the Medical Ethics Committee at the NINH, China CDC. We obtained written consent forms from all mothers.

Statistical Analysis

For peer review only - http://bmjopen.bmj.com/site/about/guidelines.xhtml

We used the past 24 hours infant and young child feeding indicator method recommended by the World Health Organization (WHO) to assess the feeding practice of 0-5 month-old-infants to generate internationally comparable rates of exclusive breastfeeding. We defined the practice of being fed exclusively with breast milk in the last 24-hours as *exclusive breastfeeding*. Even though previous studies indicated that providing prelacteal feeds is a long-held tradition in many parts of China, regional disparities were quite large [23]. Additionally, water is the top one first drink received by the newborns in China and sometimes mothers revert to exclusive breastfeeding after breast milk came in [24]. Using the method can reduce the recall bias of mothers with older infants as well as the possibility of underestimating exclusive breastfeeding[24].

We first used descriptive analysis to report the selected characteristics, and then assessed their differences between migrant and non-migrant mothers, using Pearson's Chi-square test. Potential predictors were selected through reviewing relevant literature[3,12–15,18,25]. Chi-square tests were then used to examine the potential predictors of EBF for migrant and non-migrant mothers, respectively.

Socio-demographic characteristics included maternal age (<=25 years old, 26-35 years old and >=36 years old), maternal ethnicity (Han and others), maternal and paternal education (junior high school or less, high school, and college or higher education), maternal and paternal occupation (unemployed, agriculture-related or casual work, industry or business-related, professionals or white-collars), employment type of mothers (formal and informal) and place of residence (big cities, and small and medium cities). Obstetrical history comprised parity (1 and >=2), premature birth (yes and no), low birthweight (yes and no), mode of delivery (vaginal and cesarean) and delivery hospital type (hospitals at the municipal level or above level, hospitals at the

For peer review only - http://bmjopen.bmj.com/site/about/guidelines.xhtml

district/county level and others). Breastfeeding-related factors included early initiation of breastfeeding (EIBF) (yes and no), knowledge on EBF (yes and no), low intention of breastfeeding within 1 month postpartum (yes and no), and postpartum breastfeeding difficulties (yes and no). We defined EIBF as the practice of putting infants to the breast of their mothers within the first hour of birth based on the definition of the WHO [26]. We defined mothers who were always, very often or sometimes unwilling to breastfeed within 1 month postpartum as having a low intention of breastfeeding within 1 month postpartum. Social influence denoted whether her partner, friend, mother or mother-in-law agreed that breastmilk was better than breastmilk substitutes. Health-seeking behaviors comprised the timing of considering how to feed the infants and attending antenatal visits, breastfeeding education sessions or mother groups.

We then performed logistic regressions to identify predictors of EBF. The selection of covariate variables was based on the *p*-values of Chi-square tests ($P<0.05$). Odds ratios and 95% confidence intervals were displayed. The data analyses were performed by Stata version 15.0 (Stata Corporation, USA). We set the level of statistical significance at $\alpha=0.05$.

For peer review only

Results

Sample description and factors associated with EBF

Considering that migrants usually concentrate in urban areas, we only focused on 3,571 mothers of 0-5 month-old-infants who lived in big, medium, and small cities. Migrant mothers were defined as those who had resided in a specific county for a month or more and had been in a place different from their registered county. We excluded 172 observations due to the missing values of potential predictors, which left 3,399 (>95% of the original sample) observations for statistical analysis, of which 2,199 were non-migrants and 1,200 were migrants. A sensitivity analysis using the full sample revealed very similar migrant distribution to the one shown in this study.

Table 1 displayed the summary statistics and results of the bivariate analysis, reflecting significant differences in the distribution of selected characteristics and associated factors of EBF among non-migrant and migrant mothers. The prevalence of EBF was 30.3% among non-migrant mothers, which was not statistically different from 29.0% migrant mothers (P=0.433).

Migrant mothers were statistically different from non-migrant mothers in age, ethnicity, occupation (her and her partner's), formal employment status, and place of residence. More than 60% of mothers were aged 26-35 years in both groups, while the proportion of mothers above 36 years was significantly higher in non-migrant mothers (P<0.001). The likelihood of being of Han ethnicity, working as a professional or white-collar worker, and being formally employed was statistically higher in non-migrant mothers than migrant mothers as well (Table 1). Partners of non-migrant mothers were more likely to work as professionals or white-collar workers. For non-migrant mothers, socio-demographic characteristics like maternal education and occupation, paternal education, employment status of mothers, and place of residence were significantly

For peer review only - http://bmjopen.bmj.com/site/about/guidelines.xhtml

associated with EBF (P<0.001). For migrant mothers, in addition to maternal education and occupation, paternal education, employment status of mothers, and place of residence, the ethnicity of mothers also significantly affected the prevalence of EBF. Mothers or their partners with a college or higher education were more likely to practice EBF in both groups. The prevalence of EBF was higher in mothers who had a job, were formally employed, and lived in big cities among both migrant and non-migrant mothers. Mothers from the Han ethnic group were more likely to practice EBF than mothers from minority groups among the migrant group (Table 1).

Among factors about obstetrical history, the percentage of having only one child among migrant mothers was statistically higher than for non-migrant mothers (P<0.001). The proportions of premature birth and low birthweight, were quite similar between migrant and non-migrant mothers, while the rate of cesarean section was statistically higher in non-migrant mothers than migrant mothers. Cesarean section was significantly associated with a lower prevalence of EBF for both migrant and non-migrant mothers. However, for migrant women, premature birth and low birthweight of infants were also negatively related to EBF. A significantly larger proportion of migrant mothers delivered their infants in hospitals at the municipal level or above (P<0.001). Mothers who delivered infants in hospitals at the district/county level were less likely to practice breastfeeding in both groups.

EIBF, having a low intention of breastfeeding within 1 month postpartum and breastfeeding difficulties postpartum, occurred more frequently among migrant mothers than non-migrant mothers, while only the prevalence of having breastfeeding difficulties postpartum was statistically different between the two groups. Unlike the non-migrant group where EIBF was associated with EBF significantly (P<0.001), migrant mothers were more likely to practice EBF if they had breastfeeding difficulties

For peer review only - http://bmjopen.bmj.com/site/about/guidelines.xhtml

postpartum. More than half of the mothers knew the definition of EBF, and around 10% of them had a low intention of breastfeeding within 1 month postpartum in both groups. Mothers in both groups were more likely to practice EBF if they knew its definition and were less likely to do it if their intentions of breastfeeding were low within 1 month postpartum.

Even though more than 80% of mothers received positive influence from their partners, friends, mothers or mothers-in-law about breastmilk being better than breastmilk substitutes, these influences differed among migrant and non-migrant mothers. The influence from partner and friends was significantly associated with EBF for non-migrant mothers, while the influence from their partners and their own mothers were significantly associated with EBF for migrant mothers.

Non-migrant mothers were more likely to adopt health-seeking behaviors like considering how to feed their infants before pregnancy and attending antenatal visits, breastfeeding education sessions, and mother groups, than migrant mothers. Only the timing of considering how to feed their infants was statistically different between the two groups. Migrant mothers who considered how to feed their infants before pregnancy were more likely to practice EBF. Attending breastfeeding education sessions significantly affected the EBF practice for non-migrant mothers only.

For peer review only - http://bmjopen.bmj.com/site/about/guidelines.xhtml

Table 1 Factors associated with EBF practice among mothers of 0-5 month-old-infants by migration status

	Non-migrant mothers (n=2199)					Migrant mothers (n=1200)					
	Total		EBF			Total		EBF			
	N	%	n	%	*P*-value[b]	N	%	n	%	*P*-value[b]	*P*-value[a]
Socio-Demographic characteristics											
Maternal age					0.740					0.536	<0.001
<=25 years old	448	20.4	129	28.8		283	23.6	83	29.3		
26-35 years old	1411	64.2	432	30.6		799	66.6	236	29.5		
36+ years old	340	15.5	105	30.9		118	9.8	29	24.6		
Maternal ethnicity					0.080					0.019	0.002
Others	360	16.4	123	34.2		248	20.7	57	23.0		
Han	1839	83.6	543	29.5		952	79.3	291	30.6		
Maternal education					<0.001					<0.001	0.489
Junior high school or less	666	30.3	167	25.1		361	30.1	79	21.9		
High school	422	19.2	106	25.1		212	17.7	52	24.5		
College or higher education	1111	50.5	393	35.4		627	52.3	217	34.6		
Maternal occupation					<0.001					0.001	<0.001
Unemployed	776	35.3	201	25.9		449	37.4	101	22.5		
Agriculture-related or casual work	370	16.8	101	27.3		211	17.6	70	33.2		
Industry or business-related	325	14.8	106	32.6		237	19.8	82	34.6		
Professionals or white-collars	728	33.1	258	35.4		303	25.3	95	31.4		
Paternal education					<0.001					<0.001	0.211
Junior high school or less	696	31.7	174	25.0		345	28.8	80	23.2		
High school	404	18.4	90	22.3		233	19.4	54	23.2		
College or higher education	1099	50.0	402	36.6		622	51.8	214	34.4		
Paternal occupation					0.434					0.473	<0.001
Unemployed	112	5.1	37	33.0		49	4.1	11	22.4		
Agriculture-related or casual work	597	27.1	169	28.3		317	26.4	101	31.9		
Industry or business-related	660	30.0	195	29.5		453	37.8	127	28.0		
Professionals or white-collars	830	37.7	265	31.9		381	31.8	109	28.6		
Mother was formally employed					<0.001					<0.001	0.021
No	1356	61.7	365	26.9		788	65.7	197	25.0		
Yes	843	38.3	301	35.7		412	34.3	151	36.7		
Place of residence					<0.001					<0.001	<0.001

For peer review only

Small and medium sized cities	1222	55.6	299	24.5		411	34.3	82	20.0		
Big cities	977	44.4	367	37.6		789	65.8	266	33.7		
Obstetrical history											
Parity					0.875					0.105	<0.001
>=2	1181	53.7	356	30.1		509	42.4	135	26.5		
1	1018	46.3	310	30.5		691	57.6	213	30.8		
Premature birth					0.877					0.006	0.430
No	2112	96.0	639	30.3		1159	96.6	344	29.7		
Yes	87	4.0	27	31.0		41	3.4	4	9.8		
Low birthweight					0.389					0.019	0.807
No	2104	95.7	641	30.5		1146	95.5	340	29.7		
Yes	95	4.3	25	26.3		54	4.5	8	14.8		
Mode of delivery					<0.001					0.016	0.010
Vaginal	1329	60.4	438	33.0		779	64.9	244	31.3		
Cesarean	870	39.6	228	26.2		421	35.1	104	24.7		
Delivery hospital type					0.103					0.095	<0.001
Hospitals at the municipal or above level	1348	61.3	423	31.4		849	70.8	259	30.5		
Hospitals at the district/county level	796	36.2	222	27.9		316	26.3	77	24.4		
Others	55	2.5	21	38.2		35	2.9	12	34.3		
Breastfeeding related factors											
Early initiation of breastfeeding					<0.001					0.098	0.216
No	1917	87.2	538	28.1		1028	85.7	289	28.1		
Yes	282	12.8	128	45.4		172	14.3	59	34.3		
Knowledge on EBF					<0.001					<0.001	0.708
No	940	42.7	238	25.3		505	42.1	98	19.4		
Yes	1259	57.3	428	34.0		695	57.9	250	36.0		
Low intention of breastfeeding within 1 month postpartum					<0.001					0.029	0.115
No	2018	91.8	635	31.5		1082	90.2	324	29.9		
Yes	181	8.2	31	17.1		118	9.8	24	20.3		
Breastfeeding difficulties postpartum					0.436					<0.001	0.022
No	1567	71.3	467	29.8		810	67.5	210	25.9		
Yes	632	28.7	199	31.5		390	32.5	138	35.4		
Social influence											
Partner supports breastfeeding					0.012					0.031	0.868

For peer review only

No	270	12.3	64	23.7		145	12.1	31	21.4		
Yes	1929	87.7	602	31.2		1055	87.9	317	30.0		
Friend supports breastfeeding					0.031					0.217	0.719
No	318	14.5	80	25.2		179	14.9	45	25.1		
Yes	1881	85.5	586	31.2		1021	85.1	303	29.7		
Mother supports breastfeeding					0.295					0.014	0.259
No	255	11.6	70	27.5		155	12.9	32	20.6		
Yes	1944	88.4	596	30.7		1045	87.1	316	30.2		
Mother-in-law supports breastfeeding					0.740					0.198	0.114
No	312	14.2	92	29.5		147	12.3	36	24.5		
Yes	1887	85.8	574	30.4		1053	87.8	312	29.6		
Health-seeking behaviors											
Timing of considering how to feed the infants					0.490					0.002	<0.001
Before pregnancy	1208	54.9	365	30.2		532	44.3	178	33.5		
During pregnancy	646	29.4	188	29.1		429	35.8	119	27.7		
After birth	345	15.7	113	32.8		239	19.9	51	21.3		
Attend Antenatal visits					0.992					0.243	0.067
No	89	4.0	27	30.3		65	5.4	23	35.4		
Yes	2110	96.0	639	30.3		1135	94.6	325	28.6		
Attend breastfeeding education sessions					0.046					0.249	0.540
No	1101	50.1	312	28.3		614	51.2	169	27.5		
Yes	1098	49.9	354	32.2		586	48.8	179	30.5		
Attend mother groups					0.082					0.999	0.342
No	1373	62.4	434	31.6		769	64.1	223	29.0		
Yes	826	37.6	232	28.1		431	35.9	125	29.0		

[a] *P*-value of chi-square test to compare distribution of covariate variables between migrant and non-migrant mothers.
[b] *P*-value of chi-square test to identify risk factors of exclusive breastfeeding practice among migrant and non-migrant mothers respectively.

For peer review only

Predictors of breastfeeding practice among migrant and non-migrant mothers

We then performed logistic regression to determine the predictors of EBF, respectively. We only kept covariates with *p*-values smaller than 0.05 in Table 1.

Among socio-demographic factors, place of residence was significantly associated with EBF. Migrants living in big cities were more likely to practice EBF than those in small or medium cities (adjusted OR(AOR) =1.68, 95% CI 1.20 - 2.34) after adjusting for other potential confounders. The odds of EBF were lower among migrant mothers with preterm infants (AOR= 0.27, 95% CI 0.09 - 0.81) and having a low intention of breastfeeding in the first month postpartum (AOR= 0.59, 95% CI 0.36 - 0.97). Migrant mothers who worked in agriculture-related fields or as casual workers (AOR=1.77, 95% CI 1.18 - 2.64), and who knew EBF (AOR= 2.00, 95% CI 1.51 - 2.65) were more likely to practice EBF (Table 2).

Among non-migrant mothers, we found that residence in big cities (AOR=1.40, 95% CI 1.13 - 1.73), practicing EIBF (AOR=1.78, 95% CI 1.35 - 2.33), and knowledge on EBF (AOR=1.25, 95% CI 1.02 - 1.53), were significantly associated with optimal breastfeeding practice. However, cesarean section (AOR=0.74, 95% CI 0.60 - 0.89) and low intention of breastfeeding (AOR=0.46, 95% CI 0.31 - 0.70) significantly decreased the likelihood to practice EBF (Table 3).

For peer review only - http://bmjopen.bmj.com/site/about/guidelines.xhtml

Table 2 Predictors of EBF practice among migrant mothers of 0-5 month-old-infants (n=1200)

Risk factors	AOR	95% CI
Maternal ethnicity		
Others	1	
Han	1.14	(0.79 - 1.65)
Maternal education		
Junior high school or less	1	
High school	1.15	(0.72 - 1.83)
College or higher education	1.38	(0.83 - 2.28)
Maternal occupation		
Unemployed	1	
Agriculture-related or casual work	1.77**	(1.18 - 2.64)
Industry or business related	1.25	(0.84 - 1.86)
Professionals or white-collars	0.98	(0.61 - 1.55)
Paternal education		
Junior high school or less	1	
High school	0.71	(0.45 - 1.13)
College or higher education	0.90	(0.55 - 1.47)
Mather was formally employed		
No	1	
Yes	1.29	(0.88 - 1.91)
Place of residence		
Small and medium cities	1	
Big cities	1.68**	(1.20 - 2.34)
Premature birth		
No	1	
Yes	0.27*	(0.09 - 0.81)
Low birthweight		
No	1	
Yes	0.66	(0.29 - 1.52)
Mode of delivery		
Vaginal	1	
Cesarean	0.86	(0.65 - 1.14)
Knowledge on EBF		
No	1	
Yes	2.00***	(1.51 - 2.65)
Low intention of breastfeeding within 1 month postpartum		
No	1	
Yes	0.59*	(0.36 - 0.97)
Breastfeeding difficulties postpartum		
No	1	
Yes	1.32	(0.99 - 1.75)
Partner supports breastfeeding		
No	1	
Yes	1.34	(0.77 - 2.35)
Grandmother supports breastfeeding		
No	1	
Yes	1.51	(0.88 - 2.60)
Timing of considering how to feed the infants		
Before pregnancy	1	
During pregnancy	0.87	(0.65 - 1.17)
After birth	0.75	(0.51 - 1.11)

Odds ratios were adjusted for all the covariates in the model. * $P < 0.05$, ** $P < 0.01$, *** $P < 0.001$.

For peer review only

Table 3 Predictors of EBF practice among non-migrant mothers of 0-5 month-old-infants (n=2199)

Risk factors	AOR	95% CI
Maternal education		
Junior high school or less	1	
High school	0.98	(0.70 - 1.36)
College or higher education	0.98	(0.66 - 1.45)
Maternal occupation		
Unemployed	1	
Agriculture-related or casual work	1.15	(0.85 - 1.54)
Industry or business related	1.15	(0.84 - 1.59)
Professionals or white-collar	1.20	(0.85 - 1.70)
Paternal education		
Junior high school or less	1	
High school	0.81	(0.58 - 1.13)
College or higher education	1.33	(0.92 - 1.93)
Mather was formally employed		
No	1	
Yes	0.98	(0.72 - 1.35)
Place of residence		
Small and medium cities	1	
Big cities	1.40**	(1.13 - 1.73)
Mode of delivery		
Vaginal	1	
Cesarean	0.74**	(0.60 - 0.89)
Early initiation of breastfeeding		
No	1	
Yes	1.78***	(1.35 - 2.33)
Knowledge on EBF		
No	1	
Yes	1.25*	(1.02 - 1.53)
Low intention of breastfeeding within 1 month postpartum		
No	1	
Yes	0.46***	(0.31 - 0.70)
Partner supports breastfeeding		
No	1	
Yes	1.22	(0.84 - 1.78)
Friend supports breastfeeding		
No	1	
Yes	1.14	(0.81 - 1.61)
Attend breastfeeding education sessions		
No	1	
Yes	1.11	(0.92 - 1.36)

Odds ratios were adjusted for all the covariates in the model. * $P < 0.05$, ** $P < 0.01$, *** $P < 0.001$.

For peer review only

Discussion

In this study, we found that breastfeeding practice was suboptimal in both migrant and non-migrant mothers. The prevalence of EBF among non-migrant women was not significantly different from that of migrant mothers. This is the first and largest study to explore and compare breastfeeding practices and their potential predictors among migrant and non-migrant mothers in China. Multivariate regression results suggested that similar predictors of EBF for mothers in both groups included residence in big cities, knowledge on EBF, and low intention of breastfeeding in the first month postpartum. Mothers in big cities were approximately 1.5 times more likely to practice EBF. Among migrant mothers, having premature infants and working in agriculture-related fields or as casual workers, were associated with EBF practice. However, for non-migrant mothers, EIBF and cesarean delivery were unique predictors of EBF practice.

Living in big cities was the only common variable in demographic characteristics that were significantly associated with EBF practice in both groups. Previous literature also suggested a higher rate of EBF in big cities than in middle and small cities in China using nationally representative data derived from the Chinese National Nutrition and Health Survey conducted in 2013[3]. This is possibly due to better knowledge and supportive environment for mothers in big cities than those living in small and medium cities regardless of their migration status. For example, the proportion of mothers who received information on encouraging breastfeeding was higher in big cities (55.3%) than in small and medium cities (44.7%), and more than 92% of them received encouragement from the delivery hospital in big cities. This revealed that both migrant and non-migrant mothers may benefit from better breastfeeding-related education and support, in big cities in China.

Another common predictor of EBF for both migrant and non-migrant mothers was knowledge on EBF. Some of the previous studies used knowledge scores on breastfeeding benefits as a predictor of breastfeeding practice, but they failed to provide consistent evidence

For peer review only - http://bmjopen.bmj.com/site/about/guidelines.xhtml

on its association with EBF[11,18]. However, we only focused on the key message that infants should be exclusively breastfed for the first 6 months. This implies that future education intervention projects should pay more attention to disseminating core messages in promoting optimal breastfeeding practices.

Additionally, we also found that having a low intention of breastfeeding in the first month postpartum significantly decreased the odds of practicing EBF for both groups. Cracked nipples or nipple pain, and insufficient breastmilk supply ranked as the top two reasons, which together accounted for around half of all the answers causing low breastfeeding intention. In alignment with previous studies, nipple pain or fissure within the first month postpartum was the commonest reason for the low intention of breastfeeding among mothers regardless of their migration status[27,28]. Given that nipple pain was often attributed to incorrect positioning and attachment[28], health care providers need to find effective measures to help establish proper positioning for mothers, good attachment of the babies to the breast as well as effective sucking. In addition, insufficient milk supply was frequently reported by previous studies as the leading cause of breastfeeding cessation or its exclusivity[29,30]. However, there is limited evidence on how to design interventions to address maternal perceptions or concerns of insufficient milk supply[31,32]. Further studies are needed to identify effective interventions regarding the maternal perception of milk supply.

In addition to the common predictors of EBF, as explained above, there were unique risk factors for migrant and non-migrant mothers, respectively. Among migrant mothers, having premature infants significantly decreased the prevalence of EBF. The EBF rate for migrant mothers with preterm infants was 9.8%, which was vastly lower than that of non-migrant mothers (31.0%). The low EBF rate could be attributable to mother-infant separation in neonatal intensive care units (NICUs) in China[33], which resulted in many difficulties and challenges for mothers with premature infants. The disparity in the EBF rate of premature

For peer review only

infants may come from the different choices of hospitalization services between migrant and non-migrant mothers. Even though migrant mothers are more likely to deliver their infants in higher level hospitals in general, the proportion of migrant mothers with premature infants who delivered in hospitals at the municipal or higher levels was 65.9%, lower than that of non-migrant mothers (73.6%). In China, high-level hospitals are believed to have well-trained health workers and high-quality medical care [34]. An internationally well-known practice such as Kangaroo Care (KC), has only been implemented in some NICUs of high-level hospitals in China as pilot studies, for promoting the development of premature infants [35]. Implementation of KC was hindered by the limits on parental visitation, inconsistent guidelines, and safety fears of the medical staff [35]. Medical practice in NICUs may need to encourage more involvement of parents to care for their infants.

Additionally, migrant mothers working in agriculture-related fields or as casual workers were more likely to practice EBF. This is possibly because when compared with mothers working in other fields, they have more flexible time, and the shortest commuting time of 29-minutes for those who have already gone back to work. When compared with unemployed mothers, these migrant mothers were more likely to adopt health-seeking behaviors like starting to consider how to feed their infants before pregnancy and attending breastfeeding education sessions in hospitals. These findings were in alignment with previous studies, finding that mothers who worked full-time were less likely to initiate or continue breastfeeding[36]. Our results suggested that working was not necessarily a barrier to breastfeeding. Allowing lactating mothers to work with a flexible schedule that may provide support for breastfeeding.

Among non-migrant mothers, EIBF and cesarean section were distinctive predictors of EBF. The rate of EIBF was 12.8% in non-migrant mothers, which was a little higher than the 9% found in a study in the Sichuan province[37], but much lower than the national

For peer review only - http://bmjopen.bmj.com/site/about/guidelines.xhtml

prevalence of 28.7% in 2013[38] and the 59.4% found in a study in central and western China[39]. A large regional disparity of EIBF in China may be due to the delayed process of implementing Early Essential Newborn Care (EENC) in China. Even in places where EENC had been implemented, skin-to-skin contact was often interrupted due to inadequate facilities and early initiation of breastfeeding was difficult to be ensured[40]. Hospitals in China need to speed up their process of adopting EENC recommended practices and national policies are needed to be established to help hospitals and health workers overcome obstacles in implementing these practices.

For obstetrical history-related factors, we also found that the prevalence of cesarean delivery was high and negatively associated with EBF among non-migrant mothers only. The prevalence of cesarean delivery was 39.6% among non-migrant mothers, which was higher than the national average of 36.7% in 2018[41]. Even though China had made efforts in decreasing cesarean delivery in the past decade[42], the national prevalence is still far higher than the suggested prevalence of 10-15% by the WHO[43]. The government and health facilities need to make effective policies and measures in addressing non-clinical reasons for preferring a cesarean delivery, such as maternal request and perceived convenience[44].

This study derived data from a large-scale cross-sectional survey covering 8 sample sites in urban areas, which is the first and largest study to explore and compare breastfeeding practices and their potential predictors among migrant and non-migrant mothers in China. Findings in this study filled the knowledge gap in the similarities and differences in the predictors of EBF among migrant and non-migrant mothers, which had important implications for future studies as well as health interventions to promote optimal breastfeeding practices in China.

However, our study still faced the following limitations. First, differences in the predictors of EBF among migrant and non-migrant mothers revealed in this study, may come

For peer review only - http://bmjopen.bmj.com/site/about/guidelines.xhtml
For peer review only

from different choices in hospitalization services. Migrant mothers may have restricted access to better health services due to social exclusions, lack of social networks, and health insurance. Future studies need to take a closer look at the differences in health facility choices among migrant and non-migrant mothers and identify potential obstacles faced by the migrants. Second, mothers covered in this study may not be representative of the mothers in general. That is because we only interviewed mothers who brought their infants into immunization clinics at the time of the interview, which naturally excluded children who were left behind in their early childhood and may overestimate the prevalence of EBF for both groups [45,46]. A low rate of EBF would be really problematic since it will not only result in nutrition-related harm to the early childhood development of children, but also increase the health risks of mothers in China[1]. Third, when selecting sample sites in the first stage, we considered the executive capacities, and collaboration of the provincial level CDC. Thus, sample sites selected in this study to some extent reflected a higher efficiency of health systems at the county/district level. Caution is needed when interpreting these results and the results disclosed in this study cannot be generalized to the whole urban areas of China. Fourth, responses of the mothers may be influenced by social desirability bias since data collection occurred in community health centers, where mothers usually received education on EBF.

To conclude, this study found that there was no significant difference in the prevalence of EBF between migrant and non-migrant mothers in our sampled urban areas of China. Even though the predictors of EBF shared some similarities between migrant and non-migrant mothers, future interventions may still need to adopt different strategies in promoting optimal breastfeeding practices in different groups.

For peer review only

For peer review only - http://bmjopen.bmj.com/site/about/guidelines.xhtml

Reference

1 Victora CG, Bahl R, Barros AJD, *et al.* Breastfeeding in the 21st century: epidemiology, mechanisms, and lifelong effect. *Lancet* 2016;**387**:475–90. doi:10.1016/S0140-6736(15)01024-7

2 Center for Health Statistics and Information at Ministry of Health of People's Republic of China. *An Analysis Report of National Health Services Survey in China,2008*. Beijing: 2009.

3 Duan Y, Yang Z, Lai J, *et al.* Exclusive Breastfeeding Rate and Complementary Feeding Indicators in China: A National Representative Survey in 2013. *Nutrients* 2018;**10**:249. doi:10.3390/nu10020249

4 Wang X-R, Hui EC-M, Sun J-X. Population migration, urbanization and housing prices: Evidence from the cities in China. *Habitat Int* 2017;**66**:49–56. doi:10.1016/j.habitatint.2017.05.010

5 Song Y, Sun W. Health Consequences of Rural-to-Urban Migration: Evidence from Panel Data in China. *Health Econ* 2016;**25**:1252–67. doi:10.1002/hec.3212

6 Peng Y, Chang W, Zhou H, *et al.* Factors associated with health-seeking behavior among migrant workers in Beijing, China. *BMC Health Serv Res* 2010;**10**:69. doi:10.1186/1472-6963-10-69

7 Zhao Q, Kulane A, Gao Y, *et al.* Knowledge and attitude on maternal health care among rural-to-urban migrant women in Shanghai, China. *BMC Womens Health* Published Online First: 2009. doi:10.1186/1472-6874-9-5

8 Zhao Q, Huang ZJ, Yang S, *et al.* The utilization of antenatal care among rural-to-urban migrant women in Shanghai: A hospital-based cross-sectional study. *BMC Public Health* Published Online First: 2012. doi:10.1186/1471-2458-12-

For peer review only - http://bmjopen.bmj.com/site/about/guidelines.xhtml

1012

9 WEI X, PEARSON S, ZHANG Z, *et al.* COMPARING KNOWLEDGE AND USE OF HEALTH SERVICES OF MIGRANTS FROM RURAL AND URBAN AREAS IN KUNMING CITY, CHINA. *J Biosoc Sci* 2010;**42**:743–56. doi:10.1017/S0021932010000313

10 Dennis C, Shiri R, Brown HK, *et al.* Breastfeeding rates in immigrant and non-immigrant women: A systematic review and meta-analysis. *Matern Child Nutr* 2019;**15**:e12809. doi:10.1111/mcn.12809

11 Gao H, Wang Q, Hormann E, *et al.* Breastfeeding practices on postnatal wards in urban and rural areas of the Deyang region, Sichuan province of China. *Int Breastfeed J* 2016;**11**:11. doi:10.1186/s13006-016-0070-0

12 Li L, Li S, Ali M, *et al.* Feeding practice of infants and their correlates in urban areas of Beijing, China. *Pediatr Int* 2003;**45**:400–6. doi:10.1046/j.1442-200X.2003.01754.x

13 Xu F, Binns C, Nazi G, *et al.* A comparison of breastfeeding among Han, Uygur and other ethnic groups in Xinjiang, PR China. *BMC Public Health* 2006;**6**:196. doi:10.1186/1471-2458-6-196

14 Zhao J, Zhao Y, Du M, *et al.* Maternal education and breastfeeding practices in China: A systematic review and meta-analysis. *Midwifery* 2017;**50**:62–71. doi:10.1016/j.midw.2017.03.011

15 Rollins NC, Bhandari N, Hajeebhoy N, *et al.* Why invest, and what it will take to improve breastfeeding practices? *Lancet* 2016;**387**:491–504. doi:10.1016/S0140-6736(15)01044-2

16 Li J, Nguyen TT, Wang X, *et al.* Breastfeeding practices and associated factors at the individual, family, health facility and environmental levels in China.

For peer review only

For peer review only - http://bmjopen.bmj.com/site/about/guidelines.xhtml

Manteral Child Nutr 2020.

17 Waits A, Guo CY, Chien LY. Evaluation of factors contributing to the decline in exclusive breastfeeding at 6 months postpartum: The 2011-2016 National Surveys in Taiwan. *Birth* Published Online First: 2018. doi:10.1111/birt.12340

18 Ling Shi, Jingxu Zhang, Yan Wang, *et al.* Breastfeeding in Rural China: Association Between Knowledge, Attitudes, and Practices. *J Hum Lact* 2008;**24**:377–85. doi:10.1177/0890334408319157

19 Qiu L, Zhao Y, Binns CW, *et al.* Initiation of breastfeeding and prevalence of exclusive breastfeeding at hospital discharge in urban, suburban and rural areas of Zhejiang China. *Int Breastfeed J* 2009;**4**:1. doi:10.1186/1746-4358-4-1

20 Su M, Ouyang YQ. Father's role in breastfeeding promotion: Lessons from a quasi-experimental trial in China. *Breastfeed Med* Published Online First: 2016. doi:10.1089/bfm.2015.0144

21 Ke J, Ouyang Y-Q, Redding SR. Family-Centered Breastfeeding Education to Promote Primiparas' Exclusive Breastfeeding in China. *J Hum Lact* 2018;**34**:365–78. doi:10.1177/0890334417737293

22 Huang P, Ren J, Liu Y, *et al.* Factors affecting breastfeeding adherence among Chinese mothers: A multicenter study. *Med (United States)* Published Online First: 2017. doi:10.1097/MD.0000000000007619

23 Tang L, Hewitt K, Yu C. Prelacteal Feeds in China. *Curr Pediatr Rev* 2012;**8**:304–12. doi:10.2174/157339612803307688

24 Greiner T. Exclusive breastfeeding: measurement and indicators. *Int Breastfeed J* 2014;**9**:18. doi:10.1186/1746-4358-9-18

25 Dennis C-L, Gagnon A, Van Hulst A, *et al.* Prediction of Duration of Breastfeeding among Migrant and Canadian-Born Women: Results from a

For peer review only - http://bmjopen.bmj.com/site/about/guidelines.xhtml

Multi-Center Study. *J Pediatr* 2013;**162**:72–9. doi:10.1016/j.jpeds.2012.06.041

26 Daelmans B, Dewey K, Arimond M. New and Updated Indicators for Assessing Infant and Young Child Feeding. *Food Nutr Bull* 2009;**30**:S256–62. doi:10.1177/15648265090302S210

27 McCann MF, Baydar N, Williams RL. Breastfeeding Attitudes and Reported Problems in a National Sample of WIC Participants. *J Hum Lact* 2007;**23**:314–24. doi:10.1177/0890334407307882

28 Kent J, Ashton E, Hardwick C, *et al.* Nipple Pain in Breastfeeding Mothers: Incidence, Causes and Treatments. *Int J Environ Res Public Health* 2015;**12**:12247–63. doi:10.3390/ijerph121012247

29 Balogun OO, Dagvadorj A, Anigo KM, *et al.* Factors influencing breastfeeding exclusivity during the first 6 months of life in developing countries: a quantitative and qualitative systematic review. *Matern Child Nutr* 2015;**11**:433–51. doi:10.1111/mcn.12180

30 Flaherman VJ, Beiler JS, Cabana MD, *et al.* Relationship of newborn weight loss to milk supply concern and anxiety: the impact on breastfeeding duration. *Matern Child Nutr* 2016;**12**:463–72. doi:10.1111/mcn.12171

31 Galipeau R, Baillot A, Trottier A, *et al.* Effectiveness of interventions on breastfeeding self-efficacy and perceived insufficient milk supply: A systematic review and meta-analysis. *Matern Child Nutr* 2018;**14**. doi:10.1111/mcn.12607

32 Wood NK, Sanders EA, Lewis FM, *et al.* Pilot test of a home-based program to prevent perceived insufficient milk. *Women and Birth* 2017;**30**:472–80. doi:10.1016/j.wombi.2017.04.006

33 Wang Y, Briere C-E, Xu W, *et al.* Factors Affecting Breastfeeding Outcomes

For peer review only

at Six Months in Preterm Infants. *J Hum Lact* 2019;**35**:80–9. doi:10.1177/0890334418771307

34 Wang H, Zhang D, Hou Z, *et al.* Association between social health insurance and choice of hospitals among internal migrants in China: a national cross-sectional study. *BMJ Open* 2018;**8**:e018440. doi:10.1136/bmjopen-2017-018440

35 Zhang Y, Deng Q, Zhu B, *et al.* Neonatal intensive care nurses' knowledge and beliefs regarding kangaroo care in China: a national survey. *BMJ Open* 2018;**8**:e021740. doi:10.1136/bmjopen-2018-021740

36 Mandal B, Roe BE, Fein SB. The differential effects of full-time and part-time work status on breastfeeding. *Health Policy (New York)* 2010;**97**:79–86. doi:10.1016/j.healthpol.2010.03.006

37 Tang L, Binns CW, Lee AH, *et al.* Low Prevalence of Breastfeeding Initiation within the First Hour of Life in a Rural Area of Sichuan Province, China. *Birth* 2013;**40**:134–42. doi:10.1111/birt.12038

38 UNICEF. Children in China: An Atlas of Social Indicators of Children in China 2018. Beijing: 2018.

39 Guo S, Fu X, Scherpbier RW, *et al.* Breastfeeding rates in central and western China in 2010: implications for child and population health. *Bull World Health Organ* 2013;**91**:322–31. doi:10.2471/BLT.12.111310

40 Qu W, Yue Q, Wang Y, *et al.* Implementation of the Early Essential Newborn Care (EENC) on neonatal outcomes in west China: an observational study. *Lancet* 2018;**392**:S54. doi:10.1016/S0140-6736(18)32683-7

41 Li H, Hellerstein S, Zhou Y, *et al.* Trends in Cesarean Delivery Rates in China, 2008-2018. *JAMA* 2020;**323**:89. doi:10.1001/jama.2019.17595

For peer review only

For peer review only - http://bmjopen.bmj.com/site/about/guidelines.xhtml

42 Liang J, Mu Y, Li X, *et al.* Relaxation of the one child policy and trends in caesarean section rates and birth outcomes in China between 2012 and 2016: observational study of nearly seven million health facility births. *BMJ* 2018;:k817. doi:10.1136/bmj.k817

43 Betran A, Torloni M, Zhang J, *et al.* WHO Statement on Caesarean Section Rates. *BJOG An Int J Obstet Gynaecol* 2016;**123**:667–70. doi:10.1111/1471-0528.13526

44 Long Q, Kingdon C, Yang F, *et al.* Prevalence of and reasons for women's, family members', and health professionals' preferences for cesarean section in China: A mixed-methods systematic review. *PLOS Med* 2018;**15**:e1002672. doi:10.1371/journal.pmed.1002672

45 Tian X, Ding C, Shen C, *et al.* Does Parental Migration Have Negative Impact on the Growth of Left-Behind Children?—New Evidence from Longitudinal Data in Rural China. *Int J Environ Res Public Health* 2017;**14**:1308. doi:10.3390/ijerph14111308

46 Zhang N, Bécares L, Chandola T. Does the timing of parental migration matter for child growth? A life course study on left-behind children in rural China. *BMC Public Health* 2015;**15**:966. doi:10.1186/s12889-015-2296-y

For peer review only

STROBE Statement—Checklist of items that should be included in reports of ***cross-sectional studies***

	Item No	Page number in manuscript	Recommendation
**Title and abstract**	1	Page 3	(*a*) Indicate the study's design with a commonly used term in the title or the abstract
		Page 3	(*b*) Provide in the abstract an informative and balanced summary of what was done and what was found
**Introduction**			
Background/rationale	2	Page 5	Explain the scientific background and rationale for the investigation being reported
Objectives	3	Page 5	State specific objectives, including any prespecified hypotheses
**Methods**			
Study design	4	Page 6	Present key elements of study design early in the paper
Setting	5	Page 6-7	Describe the setting, locations, and relevant dates, including periods of recruitment, exposure, follow-up, and data collection
Participants	6	Page 6-7	(*a*) Give the eligibility criteria, and the sources and methods of selection of participants
Variables	7	Page 8	Clearly define all outcomes, exposures, predictors, potential confounders, and effect modifiers. Give diagnostic criteria, if applicable
Data sources/ measurement	8*	Page 8	For each variable of interest, give sources of data and details of methods of assessment (measurement). Describe comparability of assessment methods if there is more than one group
Bias	9	Page 8	Describe any efforts to address potential sources of bias
Study size	10	Page 6	Explain how the study size was arrived at
Quantitative variables	11	Page 8	Explain how quantitative variables were handled in the analyses. If applicable, describe which groupings were chosen and why
Statistical methods	12	Page 8-9	(*a*) Describe all statistical methods, including those used to control for confounding
		Page 9	(*b*) Describe any methods used to examine subgroups and interactions
		Page 10	(*c*) Explain how missing data were addressed
		Not applicable	(*d*) If applicable, describe analytical methods taking account of sampling strategy
		Page 10	(*e*) Describe any sensitivity analyses
**Results**			
Participants	13*	Page 10	(a) Report numbers of individuals at each stage of study—eg numbers potentially eligible, examined for eligibility, confirmed eligible, included in the study, completing follow-up, and analysed
		Not applicable	(b) Give reasons for non-participation at each stage
		Not applicable	(c) Consider use of a flow diagram
Descriptive data	14*	Page 10-12	(a) Give characteristics of study participants (eg demographic, clinical, social) and information on exposures and potential confounders
		Not applicable	(b) Indicate number of participants with missing data for each variable of interest

For peer review only - http://bmjopen.bmj.com/site/about/guidelines.xhtml

Outcome data	15*	Page 10	Report numbers of outcome events or summary measures
Main results	16	Page 10-18	(*a*) Give unadjusted estimates and, if applicable, confounder-adjusted estimates and their precision (eg, 95% confidence interval). Make clear which confounders were adjusted for and why they were included
		Not applicable	(*b*) Report category boundaries when continuous variables were categorized
		Not applicable	(*c*) If relevant, consider translating estimates of relative risk into absolute risk for a meaningful time period
Other analyses	17	Page 16-18	Report other analyses done—eg analyses of subgroups and interactions, and sensitivity analyses
**Discussion**			
Key results	18	Page 19	Summarise key results with reference to study objectives
Limitations	19	Page 22-23	Discuss limitations of the study, taking into account sources of potential bias or imprecision. Discuss both direction and magnitude of any potential bias
Interpretation	20	Page 19-22	Give a cautious overall interpretation of results considering objectives, limitations, multiplicity of analyses, results from similar studies, and other relevant evidence
Generalisability	21	Page 22-23	Discuss the generalisability (external validity) of the study results
**Other information**			
Funding	22	Title page	Give the source of funding and the role of the funders for the present study and, if applicable, for the original study on which the present article is based

*Give information separately for exposed and unexposed groups.

Note: An Explanation and Elaboration article discusses each checklist item and gives methodological background and published examples of transparent reporting. The STROBE checklist is best used in conjunction with this article (freely available on the Web sites of PLoS Medicine at http://www.plosmedicine.org/, Annals of Internal Medicine at http://www.annals.org/, and Epidemiology at http://www.epidem.com/). Information on the STROBE Initiative is available at www.strobe-statement.org.

For peer review only
